# A Complex Containing SNF1-Related Kinase (SnRK1) and Adenosine Kinase in Arabidopsis

**DOI:** 10.1371/journal.pone.0087592

**Published:** 2014-01-30

**Authors:** Gireesha Mohannath, Jamie N. Jackel, Youn Hyung Lee, R. Cody Buchmann, Hui Wang, Veena Patil, Allie K. Adams, David M. Bisaro

**Affiliations:** 1 Department of Molecular Genetics, Center for Applied Plant Sciences, and Center for RNA Biology, The Ohio State University, Columbus, Ohio, United States of America; 2 Department of Horticultural Biotechnology, Kyung Hee University, Yongin, Korea; University of Santiago de Compostela School of Medicine – CIMUS, Spain

## Abstract

SNF1-related kinase (SnRK1) in plants belongs to a conserved family that includes sucrose non-fermenting 1 kinase (SNF1) in yeast and AMP-activated protein kinase (AMPK) in animals. These kinases play important roles in the regulation of cellular energy homeostasis and in response to stresses that deplete ATP, they inhibit energy consuming anabolic pathways and promote catabolism. Energy stress is sensed by increased AMP:ATP ratios and in plants, 5′-AMP inhibits inactivation of phosphorylated SnRK1 by phosphatase. In previous studies, we showed that geminivirus pathogenicity proteins interact with both SnRK1 and adenosine kinase (ADK), which phosphorylates adenosine to generate 5′-AMP. This suggested a relationship between SnRK1 and ADK, which we investigate in the studies described here. We demonstrate that SnRK1 and ADK physically associate in the cytoplasm, and that SnRK1 stimulates ADK *in vitro* by an unknown, non-enzymatic mechanism. Further, altering SnRK1 or ADK activity in transgenic plants altered the activity of the other kinase, providing evidence for *in vivo* linkage but also revealing that *in vivo* regulation of these activities is complex. This study establishes the existence of SnRK1-ADK complexes that may play important roles in energy homeostasis and cellular responses to biotic and abiotic stress.

## Introduction

The evolutionarily conserved SNF1/AMPK/SnRK1 family of protein kinases includes SNF1 kinase (sucrose non-fermenting 1) in yeast, AMPK (AMP-activated protein kinase) in animals, and SnRK1 (SNF1-related kinase 1) in plants. These serine/threonine kinases play a central role in the regulation of metabolism by responding to cellular energy charge, as sensed by relative AMP and ATP concentrations [1]–[5]. Due to the action of adenylate kinase, nutritional and environmental stresses that deplete ATP lead to increased AMP levels, and when the AMP:ATP ratio is elevated the SNF1/AMPK/SnRK1 kinases turn off energy-consuming biosynthetic pathways, promote the accumulation of storage carbohydrate, and turn on alternative ATP-generating systems. For example, in plants, SnRK1 phosphorylates and inactivates key enzymes that control steroid and isoprenoid biosynthesis, nitrogen assimilation for amino acid and nucleotide synthesis, and sucrose synthesis [6]. SnRK1 also plays a role in metabolic signaling by phosphorylating trehalose-6-phosphate synthase enzymes implicated in signaling processes [7], [8]. In addition to altering enzyme activities, SnRK1 also integrates energy and stress signals by inducing extensive changes in the transcriptome that promote catabolism and inhibit anabolic pathways [9], [10]. Given their dependence on the host both for biosynthetic machinery and energy, it is not surprising that viruses have been linked to these kinases. This was first recognized in plants, where SnRK1-mediated responses were identified as a component of innate antiviral defense that is antagonized by the geminivirus pathogenicity proteins AL2 and L2, which interact with SnRK1 [11], [12]. The geminivirus Rep protein (AL1) has also been shown to interact with the SnRK1 activating kinases GRIK1 and GRIK2 (geminivirus Rep interacting kinases, also known as *Arabidopsis thaliana* SnRK1 activating kinases, AtSnAK)[13]. More recently, interactions between AMPK and several mammalian viruses have been described, including HIV, SV40, hepatitis C virus, and human cytomegalovirus [14]–[17].

SNF1/AMPK/SnRK1 kinases function *in vivo* as heterotrimeric complexes comprised of an α catalytic subunit, a γ subunit, and a β subunit that bridges the α and γ subunits. Budding yeast has only a single α protein (SNF1), but multiple genes encoding α, β, and γ subunits are typical, suggesting combinatorial usage of isoforms and the potential for complex regulation [1]–[5]. The α subunits consist of an N-terminal kinase domain that contains a conserved activation loop, and a C-terminal domain required for interaction with the β and γ subunits. The γ subunits contain tandemly repeated cystathionine β-synthase (CBS) motifs, which act in pairs to form Bateman motifs that bind ATP or AMP in a mutually exclusive manner, providing a structural basis for energy sensor function [18], [19].

Mechanisms that activate SNF1/AMPK/SnRK1 complexes are not completely understood, but all are stimulated by upstream kinases that phosphorylate the catalytic α subunit in the activation loop at threonine 172 in AMPK and analogous residues in SNF1 and SnRK1 [20]–[22]. Multiple upstream activating kinases have been identified in mammals, yeast, and plants [23]–[28]. This essential phosphorylation event triggers autophosphorylation at several sites, although the consequences of this cascade are not clear. In the case of AMPK, AMP allosterically stimulates kinase activity and inhibits kinase inactivation by protein phosphatase 2C (PP2C)-catalyzed dephosphorylation of threonine 172 [29]–[31]. These effects are mediated by AMP binding to the γ subunit and are antagonized by ATP [18], [31]. Direct allosteric stimulation of SnRK1 complexes by AMP does not occur. However, AMP inhibits dephosphorylation of the activation loop threonine and subsequent inactivation of the plant enzyme [22]. Thus, while some differences in activation mechanism may exist, it is well established that AMP promotes SNF1/AMPK/SnRK1 activity while ATP has the opposite effect.

With nearly 40 members in Arabidopsis, the family of catalytic subunits in plants has diversified into three subfamilies, called SnRK1, SnRK2, and SnRK3 [3], [32]. The greatest expansion has occurred in the SnRK2 and SnRK3 subfamilies, which are unique to plants. These kinases are considerably diverged from SNF1 and AMPK and unlike SnRK1, those tested do not complement *snf1* mutants. Arabidopsis has two expressed *SnRK1* genes that encode SnRK1.1 (also called Arabidopsis kinase 10; AKIN10) and SnRK1.2 (AKIN11). Both are 512 amino acids in length, with 89% sequence identity in the N-terminal kinase domain and 64% in the C-terminal domain. They also share ∼50% overall identity with SNF1 and AMPK, with 60–65% identity in the kinase catalytic domain. The work described in this study was carried out with SnRK1.2 and its isolated kinase domain, which for simplicity are referred to as SnRK1 and SnRK1-KD, respectively.

Adenosine kinase (ADK) is a eukaryotic purine kinase that catalyzes transfer of γ-phosphate from ATP to adenosine to generate 5′-AMP. Arabidopsis encodes two ADK proteins, ADK1 and ADK2, with 92% amino acid identity [33]. The studies described here were performed with ADK2, which for convenience is referred to as ADK. ADK is important for adenosine salvage and the synthesis of nucleic acids and nucleotide cofactors. It is also involved in cytokinin regulation in plants, and in plants and yeast has been shown to maintain methyltransferase activities by sustaining the methyl cycle that generates S-adenosyl methionine [34]–[36]. Repressive DNA and histone methylation, and inactivation of the adenosine derivative cytokinin by phosphorylation, are components of the antiviral response to DNA viruses which geminivirus AL2 and L2 proteins counter by interacting with and inhibiting ADK [12], [37]–[42].

Because AMP, a product of ADK, sustains SnRK1 activity, and geminivirus pathogenicity proteins interact with both SnRK1 and ADK, we decided to explore the relationship between these kinases. Here we present several lines of evidence to demonstrate that SnRK1 and ADK form *in vivo* complexes, and that cellular SnRK1 and ADK activities are linked. In addition, we describe a novel mechanism for ADK stimulation by SnRK1 that is independent of SnRK1 kinase activity.

## Results

### SnRK1 Assay and Protein Expression

**Figure 1:**
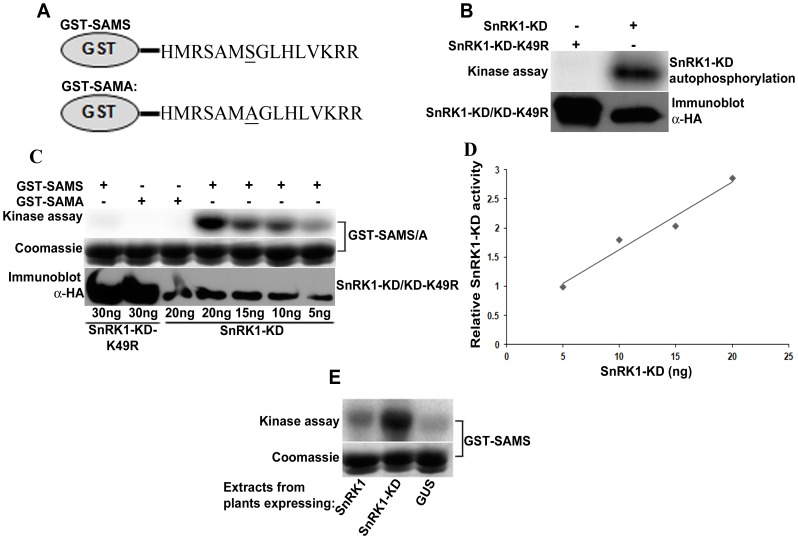
The *in vitro* SnRK1 assay. (A) Diagrams of GST-SAMS and GST-SAMA substrates. The target serine in GST-SAMS, containing a SNF1/AMPK/SnRK1 consensus site, is replaced by alanine in GST-SAMA (residues underlined). The line connecting the SAMS/SAMA sequence to GST indicates a flexible polyglycine linker. These substrates were expressed and purified from *E. coli* cells. (B) Autophosphorylation assay confirmed that SnRK1-KD, but not SnRK1-KD-K49R, expressed in *N. benthamiana* is active. HA_2_His_6_-tagged SnRK1-KD and SnRK1-KD-K49R (inactive mutant, negative control) expressed and purified from *N. benthamiana* were incubated with γ^32^P-ATP alone or with GST-SAMS or GST-SAMA. Aliquots were fractionated on polyacrylamide gels containing SDS (SDS-PAGE) and exposed to a phosphor-imager to detect labeled proteins. For loading controls, GST-SAMS and GST-SAMA were monitored by Coomassie staining, while immunoblots were probed with anti-HA (α-HA) to detect the kinases. (C) Kinase assays in which GST-SAMS or GST-SAMA (3 µg) were incubated with varying amounts of SnRK1-KD or SnRK1-KD-K49R, as indicated. (D) A linear correlation (R^2^ = 0.9664) was observed between the intensity of labeled GST-SAMS signal and the amount of SnRK1-KD added in the range tested. The data shown are representative of three experiments, and were normalized to activity observed with 5 ng SnRK1-KD. (E) Extracts from *N. benthamiana* cells expressing full-length SnRK1, SnRK1-KD, or GUS (control for endogenous activity) from a plasmid vector were assayed following the addition of γ^2^P-ATP and GST-SAMS. SnRK1-KD activity data (autophosphorylation and phosphorylation of GST-SAMS) is presented in Table S1.

Many SNF1/AMPK/SnRK1 assays rely on phosphorylation of peptides containing a consensus recognition sequence. To more simply detect and measure kinase activity, we developed a gel-based assay by fusing the SAMS peptide, which contains such a consensus, with glutathione-S-transferase to create GST-SAMS (phosphorylation site underlined) [43], [44]. A similar fusion protein with the target serine substituted by alanine (GST-SAMA) was used as a negative control (Figure 1A). Both were expressed in *E. coli* and purified by glutathione-agarose chromatography. Because it proved difficult to express full-length SnRK1, the SnRK1 kinase domain (SnRK1-KD; amino acids 1–343) was used for *in vitro* experiments. Arabidopsis SnRK1-KD, and a kinase-inactive form containing an arginine for lysine substitution in the ATP binding site (SnRK1-KD-K49R) [11], were constructed as N-terminal, double hemagglutinin peptide-six histidine (HA_2_His_6_) fusions. Proteins were expressed in *Nicotiana benthamiana* leaf cells using TRBO, a *Tobacco mosaic virus* (TMV)-based vector, following delivery of vector constructs by agroinfiltration [45], [46]. Expressed proteins were partially purified from leaf extracts by nickel-NTA chromatography (Figure S1), and autophosphorylation assays with γ^32^P-ATP showed that recombinant SnRK1-KD was active, while SnRK1-KD-K49R displayed only background activity (Figure 1B) (Table S1). While autophosphorylation by SnRK1 is a controversial issue, we routinely observe SnRK1-KD autophosphorylation following expression from virus-based constructs in insect (11) and *N. benthamiana* cells, which contain upstream activating kinases. By comparison, SnRK1-KD expressed in *E. coli* displays negligible activity (Figure S2A).

Phosphorylation of GST-SAMS and GST-SAMA substrates was tested with varying amounts of SnRK1-KD and SnRK1-KD-K49R. Kinase preparations were pre-incubated for 20 min with unlabeled ATP (0.5 mM) to allow complete autophosphorylation before the addition of substrates and γ^32^P-ATP. Pre-incubation was judged to be adequate when SnRK1-KD autophosphorylation signal was nearly undetectable after addition of labeled ATP. GST-SAMS was phosphorylated by SnRK1-KD and levels correlated with the amount of kinase added, showing that the assay is sensitive and quantitative (Figure 1C and 1D) (Table S1). By contrast, only background signal was observed with GST-SAMA, confirming that neither GST nor the SAMA peptide is a SnRK1 substrate. SnRK1-KD-K49R failed to phosphorylate either GST-SAMS or GST-SAMA above background levels, validating its use as a kinase negative control (Figure 1C). Highlighting the sensitivity of GST-SAMS, it was also possible to detect SnRK1 activity in extracts from *N. benthamiana* leaves agroinfiltrated with non-replicating plasmid vectors expressing full-length SnRK1 or SnRK1-KD from the *Cauliflower mosaic virus* (CaMV) 35S promoter, although considerably more activity was observed with the latter (Figure 1E). The basal activity observed in extracts obtained from control plants expressing β-glucuronidase (GUS) is likely due to endogenous *N. benthamiana* SnRK1.

### SnRK1 and ADK Interact in Yeast and Plant Cells

To determine whether SnRK1 and ADK can physically associate, we tested their ability to interact in the yeast two-hybrid system using strain Y190, which contains *His3* and *LacZ* reporter genes [47]. Indeed, strong interaction was indicated by robust growth in synthetic complete medium lacking histidine when cells co-expressed full-length Arabidopsis SnRK1 and ADK, or SnRK1-KD and ADK (Table 1). In all cases cells were also positive for LacZ reporter activity, confirming the interactions. The ADK-SnRK1/SnRK1-KD interactions were detected regardless of whether the proteins were expressed as bait or prey, and none were observed with negative control proteins. These results indicate that SnRK1 and ADK specifically interact, and that the N-terminal SnRK1 kinase domain (amino acids 1-343) is sufficient for interaction.

**Table 1:** ADK interacts with SnRK1 and SnRK1-KD in yeast cells.

Bait	Prey	Interaction
SnRK1	ADK	+
ADK	SnRK1	+
SnRK1-KD	ADK	+
ADK	SnRK1-KD	+
ADK	CAT	-
ADK	p53	-
SnRK1	CAT	-
SnRK1	p53	-

The indicated bait proteins were expressed as GAL4 DNA binding domain fusions, and the prey proteins as GAL4 activation domain fusions, in yeast Y190 cells. SnRK1 indicates the full-length protein and SnRK1-KD denotes the kinase domain. Chloramphenicol acetyl transferase (CAT) and p53 were negative controls. Interaction was indicated by cell growth on medium lacking histidine and containing 50 mM aminotriazole. As an additional indicator of interaction, colonies were monitored for LacZ activity (blue color) using a filter-lift assay.

Bimolecular fluorescence complementation (BiFC) of yellow fluorescent protein (YFP) was employed to examine whether and where SnRK1 and ADK interact in plant cells [48]. Constructs expressing full-length Arabidopsis SnRK1 and ADK fused to the N- or C-terminal portions of YFP (YN and YC, respectively) were introduced into *N. benthamiana* leaf cells by agroinfiltration [49]. Constructs expressing similar Dicer-like 4 and dsRNA binding protein 4 (DCL4 and DRB4) fusion proteins were used as controls. DCL4 and DRB4 are known interaction partners [50]. Cells expressing oppositely tagged proteins (i.e., YN+YC fusion proteins potentially capable of reconstituting YFP) were viewed under a confocal microscope 48 h post-infiltration. Histone 2B fused to red fluorescent protein (RFP-H2B) served as a marker for the nucleus. Test proteins were examined in all possible combinations before it was judged that a particular pair did not interact.

**Figure 2:**
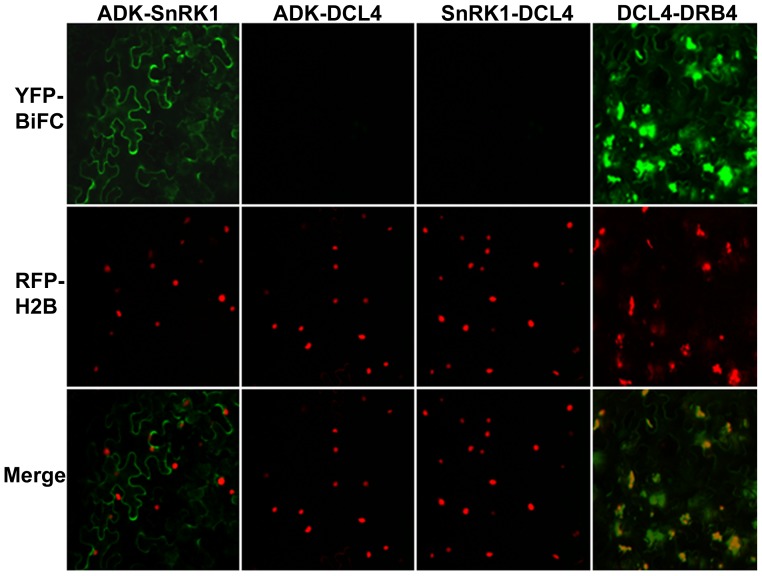
SnRK1 and ADK interact in the cytoplasm. Constructs expressing full-length SnRK1, ADK, DCL4, or DRB4 proteins fused to the N- or C-terminal portion of YFP were delivered by agroinfiltration to *N. benthamiana* leaves. Images were captured at 40x magnification 48 h post-infiltration using a confocal microscope. Representative images of epidermal cells, which have a characteristic irregular shape and a large vacuole that restricts the cytoplasm to the cell periphery, are shown. Histone H2B fused to RFP (RFP-H2B) is a marker for the nucleus.

The leaf epidermal cells observed in these experiments are irregularly shaped and contain a large central vacuole that confines the cytoplasm and nucleus to a thin strip adjacent to the cell wall. No signal was detected when only one of the fusion proteins was expressed (not shown). Additionally, there was no signal when ADK or SnRK1 were co-expressed with DCL4, although YFP fluorescence indicating DCL4:DRB4 interaction was detected in both the cytoplasm and the nucleus when these proteins were co-expressed (Figure 2). ADK is a predominantly cytoplasmic protein, whereas SnRK1 is found in the cytoplasm and the nucleus. When SnRK1 and ADK were co-expressed, strong YFP fluorescence was observed in the cytoplasm (Figure 2), indicating that these kinases form mostly extranuclear complexes *in vivo*.

### SnRK1 Co-immunoprecipitates with Endogenous ADK, and Co-purifies with Expressed ADK

**Figure 3:**
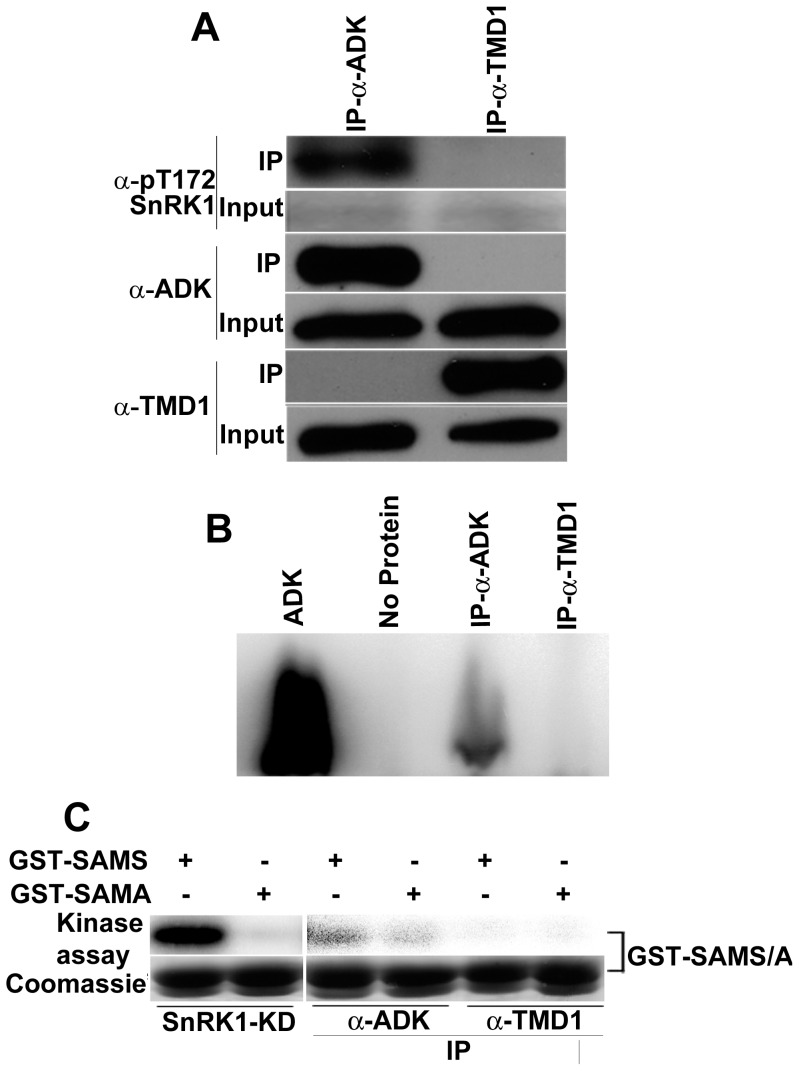
SnRK1 co-immunoprecipitates with ADK. (A) Immunoprecipitation (IP) and immunodetection were performed with ADK antibody (α-ADK) and TMD1 antibody (α-TMD1). Activated SnRK1 was detected using an antibody raised against phosphorylated AMPK (α-pT172). (B) Immunoprecipitates obtained with α-ADK or α-TMD1 were incubated with 1 µM adenosine and γ^32^P-ATP. Controls contained purified ADK or no added protein. Reaction mixtures were resolved by thin layer chromatography and labeled AMP was detected using a phosphor-imager. (C) Immunoprecipitates obtained with α-ADK or α-TMD1 were incubated with 3 µg of GST-SAMS or GST-SAMA and γ^32^P-ATP. Assays with purified SnRK1-KD were included as controls. Samples were fractionated on SDS-PAGE gels and exposed to a phosphor-imager for 2 h (SnRK1-KD controls) or 2 days (immunoprecipitate samples). GST-SAMS and GST-SAMA were monitored by Coomassie stain.

Immunoprecipitation experiments were performed to verify that SnRK1 and ADK form an *in vivo* complex. These employed a polyclonal antibody raised against Arabidopsis ADK2 (α-ADK) [37], and extracts from Arabidopsis (Col-0) plants. An antibody against phosphorylated threonine 172 in the AMPK activation loop (α-pT172) was used to detect activated SnRK1 (the corresponding residue is threonine 176). The pT172 antibody was previously shown to cross-react specifically with SnRK1 [8], [27]. SnRK1 is not abundant, and only faint signals were detected by α-pT172 in input samples. However, a clear signal corresponding to activated full-length SnRK1, which migrates with an apparent molecular weight of ∼75 kDa, was detected in immunoprecipitates obtained with α-ADK (Figure 3A). While somewhat slower than expected (calculated MW ∼59 kDa), similar anomalous migration of SnRK1 has been reported by others and may reflect post-translational modifications [51]. As expected, α-ADK also precipitated endogenous ADK protein, as shown by immunoblot and confirmed by activity assays that measure synthesis of labeled AMP from adenosine and γ^32^P-ATP (Figure 3A and 3B) [37]. By contrast, an antibody raised against Arabidopsis trans-membrane domain protein-1 (α-TMD1) failed to bring down either SnRK1 or ADK (Figure 3A and 3B). To confirm the presence of SnRK1 in immunoprecipitates, GST-SAMS or GST-SAMA was added to aliquots of immune complexes. Despite the fact that full-length SnRK1 is considerably less active than SnRK1-KD, it was possible to detect SnRK1 activity, as judged by phosphorylation of GST-SAMS, in immunoprecipitates obtained with α-ADK, but not with α-TMD1 (Figure 3C). Some background phosphorylation of GST-SAMA was also observed in α-ADK but not α-TMD immunoprecipitates, and is also apparent in the SnRK1-KD control.

**Figure 4:**
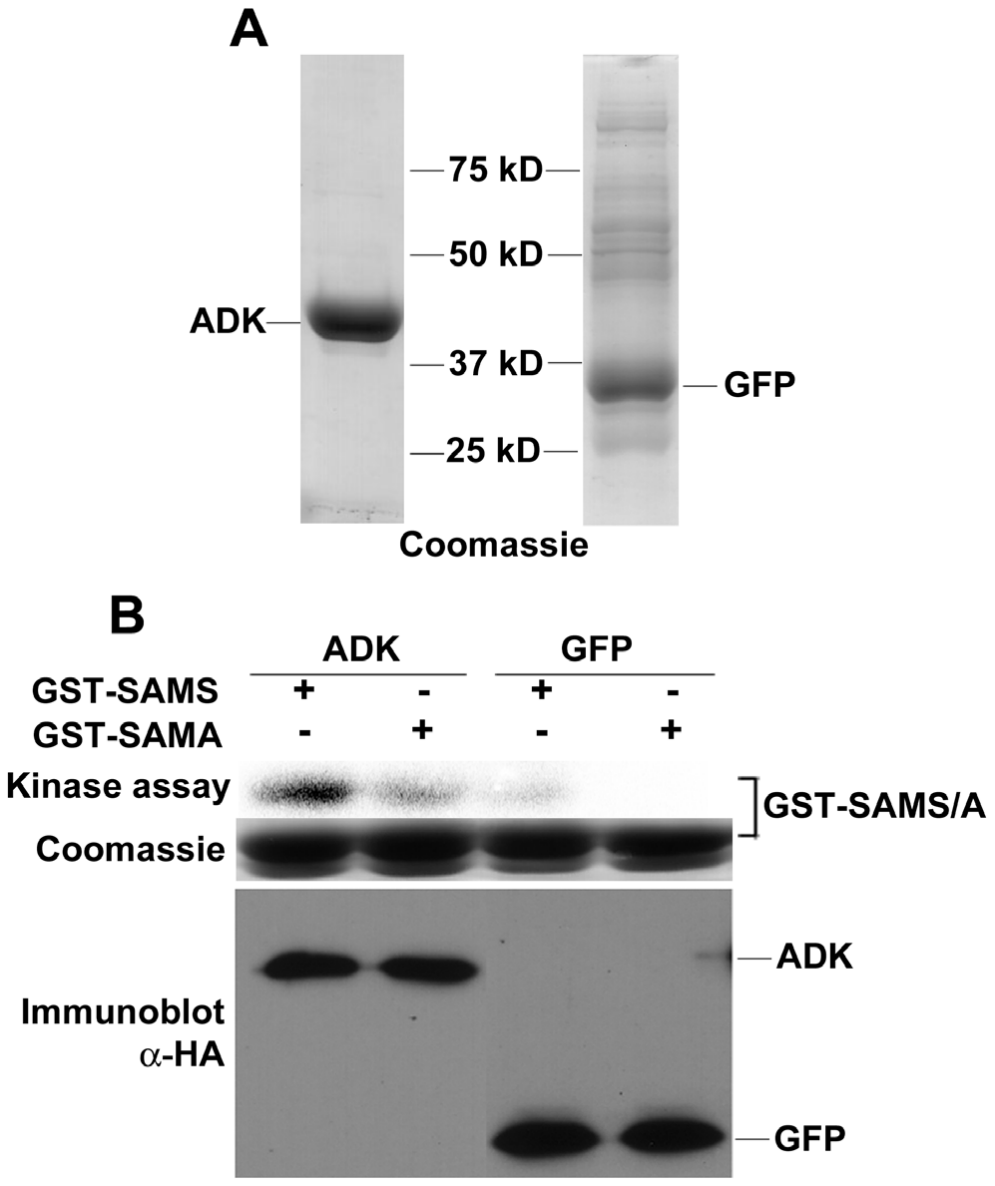
Endogenous *N. benthamiana* SnRK1 activity co-purifies with expressed ADK. (A) HA_2_His_6_-tagged Arabidopsis ADK and HA_2_His_6_-GFP (control) were expressed in *N. benthamiana* and enriched using nickel-NTA resin. Proteins were fractionated by SDS-PAGE, and Coomassie stained samples are shown. (B) GST-SAMS and γ^32^P-ATP were added to ADK or GFP preparations (3 µg protein). Samples were separated by SDS-PAGE and exposed to a phosphor-imager for 2 days. The Coomassie stained panel is a loading control for GST-SAMS and GST-SAMA, while the immunoblot (bottom panel, probed with anti-HA) is a loading control for ADK and GFP.

As an additional test of association, we asked whether SnRK1 and ADK could be co-purified. HA_2_His_6_-tagged Arabidopsis ADK was expressed in *N. benthamiana* leaf cells using the TRBO vector. Following nickel-NTA chromatography, HA_2_His_6_-ADK preparations contained a single major protein species, as seen by Coomassie stain (Figure 4A). Preparations were then tested for SnRK1 activity following addition of GST-SAMS or GST-SAMA and γ^32^P-ATP. An activity capable of phosphorylating GST-SAMS was present in HA_2_His_6_-ADK preparations, but only background signal was detected with preparations of similarly tagged, expressed, and purified green fluorescent protein (GFP) (Figure 4B). The most likely explanation for this result is that an endogenous *N. benthamiana* SnRK1 activity co-purified with the Arabidopsis ADK protein. In reciprocal experiments, we were unable to detect co-purification of endogenous ADK with expressed HA_2_His_6_-tagged SnRK1-KD or SnRK1-KD-K49R, most likely because of the much lower expression levels of these proteins from the TRBO vector (more than 50-fold less than ADK).

Together, the results of two-hybrid and BiFC studies, as well as co-immunoprecipitation and co-purification experiments, provide strong evidence for a cytoplasmic SnRK1-ADK complex that appears to be conserved between Arabidopsis and *N. benthamiana*.

### SnRK1 Phosphorylates ADK *in vitro*

Analysis of the Arabidopsis ADK1 and ADK2 sequences revealed four potential SNF1/AMPK/SnRK1 phosphorylation sites that resemble the minimal recognition motif: Hyd-(Basic/X)-X_3_-Ser/Thr-X_3_-Hyd, where Hyd indicates the hydrophobic residues M, L, V, F, or I [52]. Three of the sites are evolutionarily conserved through mono- and dicotyledonous plants and the moss *Physcomitrella patens*, while two appear to be conserved in nearly all eukaryotes, including budding yeast, fruit flies, nematodes, zebrafish, mouse, and human (Table S2). Given that deep evolutionary conservation of consensus sites is predictive of kinase-substrate relationships [53], we tested whether ADK can be phosphorylated by SnRK1.

**Figure 5:**
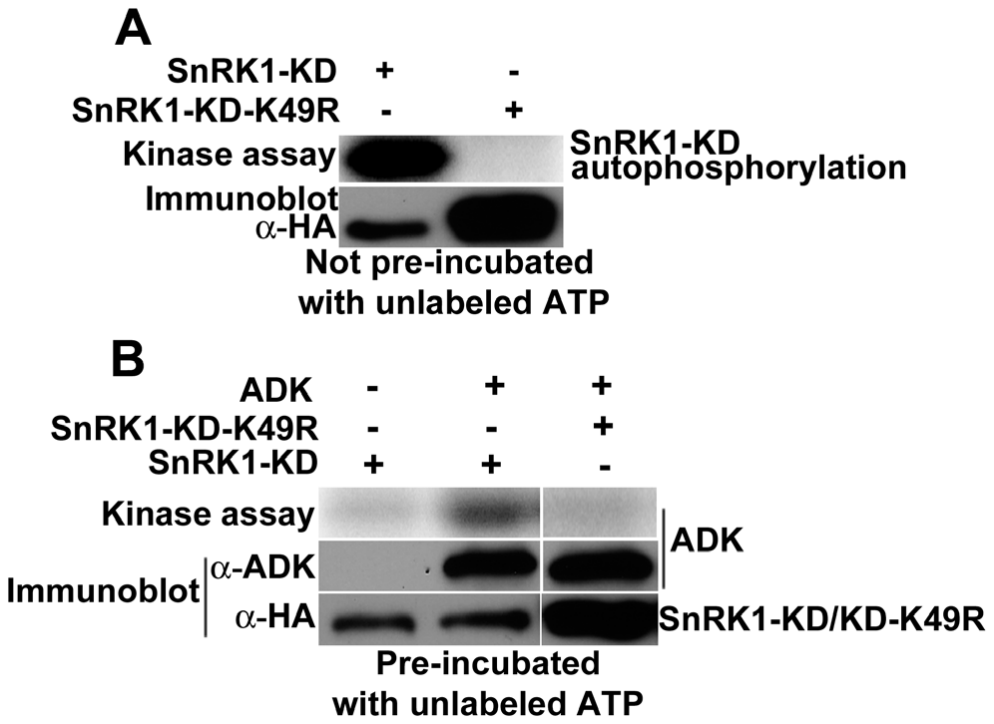
ADK is phosphorylated by SnRK1 *in vitro*. Kinase assays were conducted using γ^32^P-ATP with SnRK1-KD or SnRK1-KD-K49R either alone or with ADK. With the exception of autophosphorylation assays, SnRK1-KD and SnRK1-KD-K49R were pre-incubated with unlabeled ATP to obscure autophosphorylation. Samples were fractioned by SDS-PAGE and signals detected using a phosphor-imager. Immunoblots were probed with anti-ADK to detect ADK, or with anti-HA to detect SnRK1-KD or SnRK1-KD-K49R. (A) Kinase assays with SnRK1-KD (10 ng) and SnRK1-KD-K49R (30 ng) were performed without pre-incubation with unlabeled ATP. (B) ADK protein (3 µg) was incubated with SnRK1-KD (10 ng) or SnRK1-KD-K49R (30 ng). Note that SnRK1-KD autophosphorylation (in the lane lacking ADK) was nearly undetectable due to pre-incubation of SnRK1 with unlabeled ATP. The same pre-incubated SnRK1-KD and SnRK1-KD-K49R preparations were used to perform the GST-SAMS phosphorylation experiment shown in Figure 1C. Activity data are presented in Table S3.

Kinase assays employed SnRK1-KD, SnRK1-KD-K49R, and ADK expressed in plant cells as HA_2_His_6_ fusion proteins, and thus detectable by HA antibody. However, because these proteins migrate to nearly the same position in polyacrylamide gels, when samples were to include both SnRK1-KD (or SnRK1-KD-K49R) and ADK, aliquots were removed before ADK addition for immunoblot analysis with α-HA. ADK antibody was then used to uniquely detect ADK following the addition of this kinase. For the same reason, it was also necessary to obscure SnRK1-KD autophosphorylation. This was accomplished by pre-incubating SnRK1-KD (or SnRK1-KD-K49R) with 0.5 mM unlabeled ATP in kinase buffer for 20 min. After pre-incubation, signal due to SnRK1-KD autophosphorylation was nearly undetectable (compare left lanes of Figure 5A and 5B). Following the pre-incubation, unlabeled ATP was removed from SnRK1-KD preparations and ADK was added along with γ^32^P-ATP.

In reactions containing the pre-incubated SnRK1-KD (10 ng) and ADK, a labeled species corresponding to ADK was readily observed, whereas this band was absent in samples containing SnRK1-KD-K49R (30 ng) and ADK (Figure 5B). The same pre-incubated SnRK1-KD preparation was used with GST-SAMS to generate the data shown in Figure 1C. The ADK phosphorylation signal was about one-third that of the comparable GST-SAMS sample with 10 ng SnRK1-KD (see Fig. 1C; Table S3). Based on these results, we concluded that SnRK1 could phosphorylate ADK *in vitro.*

### SnRK1 Stimulates ADK in a Non-enzymatic Manner

Because ADK is a SnRK1 substrate, we investigated the impact of phosphorylation on ADK activity. In these experiments, which employed HA_2_His_6_ fusion proteins expressed in *N. benthamiana*, ADK was pre-incubated for 10 min with γ^32^P-ATP in the presence of a two-fold molar excess of SnRK1-KD (to permit ADK phosphorylation), SnRK1-KD-K49R, or negative control proteins. Adenosine was then added and its conversion to 5′-AMP was measured [37].

**Figure 6:**
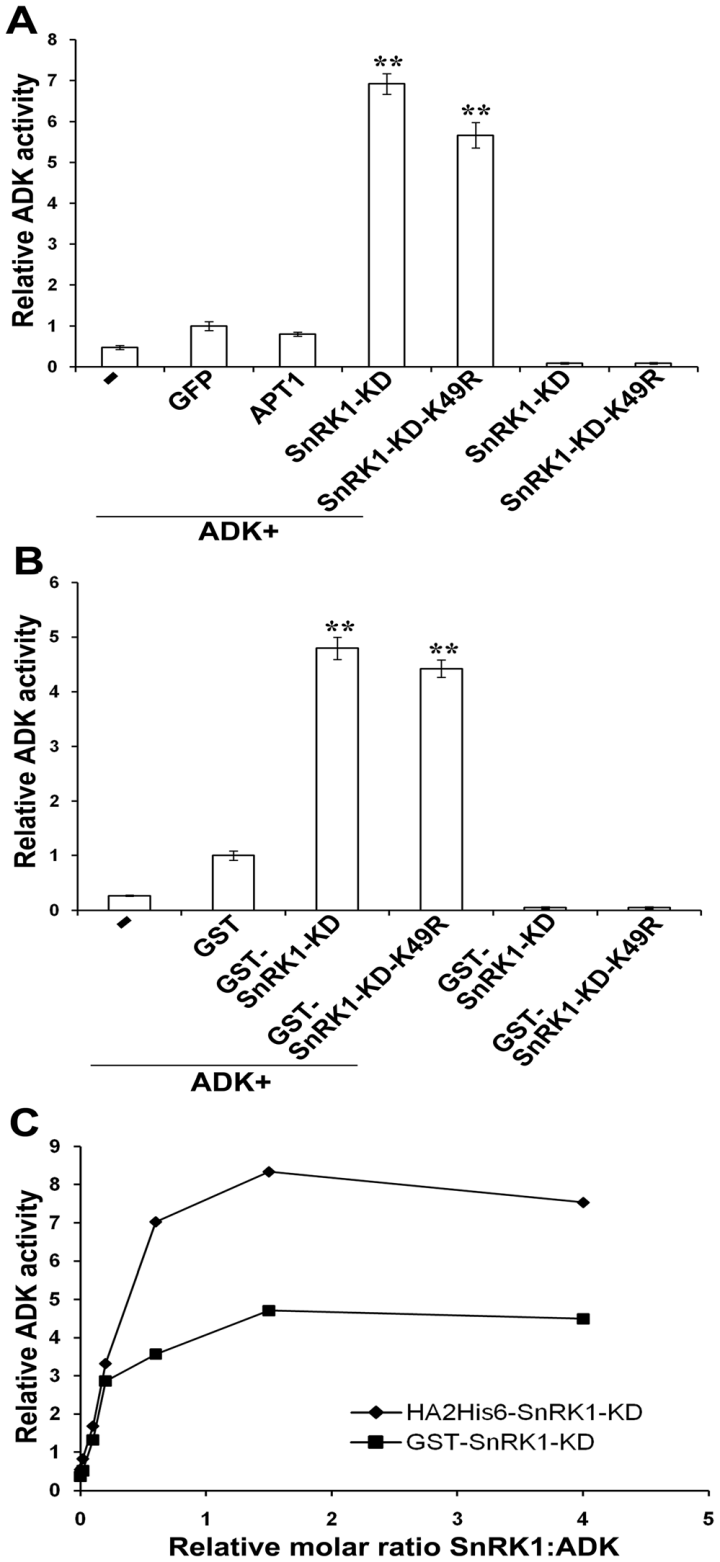
SnRK1-KD and SnRK1-KD-K49R stimulate ADK activity *in vitro*. (A) ADK (10 ng) was incubated alone, or with two-fold molar excess of HA_2_His_6_-tagged SnRK1-KD, SnRK1-KD-K49R, GFP, or APT1 purified from *N. benthamiana*. ADK activity was measured and normalized to ADK+GFP. (B) ADK (10 ng) was incubated alone, or with a two-fold molar excess of GST, GST-fused SnRK1-KD, or SnRK1-KD-K49R purified from *E. coli*. ADK activity was normalized to ADK+GST. In each case, data were obtained from three independent experiments, with two replicates each. Values indicate the mean +/− SE. Asterisks (**) indicate significant differences in ADK activity at 99% confidence, as determined by Student's *t* test. (C) The graph shows relative ADK activity plotted against increasing SnRK1-KD:ADK ratio. HA_2_His_6_-SnRK1-KD (diamonds); GST-SnRK1-KD (squares). The data are representative of three experiments. ADK activity data are presented in Table S4A-C.

No ADK activity was detected in SnRK1-KD or SnRK1-KD-K49R preparations, and only a relatively small increase in activity (two to three-fold) over reactions containing ADK alone was observed when the enzyme was incubated with the negative control proteins GFP or adenine phosphoribosyl transferase-1 (APT1). This is likely due to a non-specific, stabilizing effect of added protein. By contrast, a substantial increase in ADK activity (∼6-fold greater than ADK GFP) was observed in the presence of SnRK1-KD. Surprisingly, a comparable increase was also seen with SnRK1-KD-K49R (Figure 6A) (Table S4A). This suggested that SnRK1 stimulates ADK activity whether or not it is able to phosphorylate ADK.

Because all proteins used in these experiments, including GFP and APT1, were similarly expressed and purified from *N. benthamiana* extracts, it is unlikely that ADK stimulation was due to non-specific, contaminating proteins. However, to eliminate the possibility that unknown plant proteins that might associate and co-purify with SnRK1-KD and SnRK1-KD-K49R were responsible, these were expressed and purified from *E. coli* as GST fusion proteins. *E. coli* lacks both ADK and SnRK1-like proteins. Due to the absence of upstream activating kinases, GST-SnRK1-KD displayed extremely low autophosphorylation activity, a similarly weak ability to phosphorylate GST-SAMS, and was unable to phosphorylate ADK to a detectable extent (Figure S2; Table S5). Nevertheless, when incubated with ADK, both GST-SnRK1-KD and GST-SnRK1-KD-K49R caused increases in ADK activity comparable to the plant-expressed proteins (Figure 6B) (Table S4B).

The stoichiometry of stimulation was determined by measuring ADK activity in the presence of increasing amounts of SnRK1-KD. Using proteins expressed in *N. benthamiana*, ADK activity reached ∼90% of maximum at an approximate 1∶1 molar ratio of HA_2_His_6_-SnRK1-KD:ADK, and no significant further increase was observed at molar ratios as high as 4∶1. A similar outcome was obtained when this experiment was repeated using GST-SnRK1-KD expressed in *E. coli* (Figure 6C) (Table S4C).

We then considered the opposite question, namely whether ADK or its product could modulate SnRK1-KD activity *in vitro*. However, neither AMP (50–500 µM) nor a two-fold molar excess of ADK increased SnRK1-KD activity, as determined by kinase assays with GST-SAMS (Figure S3). This is consistent with the concept that AMP does not allosterically activate SnRK1, but rather inhibits its inactivation by a phosphatase [22].

The results of these studies conclusively demonstrate that SnRK1-KD and ADK physically associate, and indicate that a functional outcome of this is a significant stimulation of ADK activity. Further, SnRK1 phosphorylation of ADK is not necessary for the increase in activity, which instead appears to be a result of direct, stoichiometric interaction between the two proteins. Thus, while there may be *in vivo* consequences of ADK phosphorylation by SnRK1, there is no apparent *in vitro* effect.

### Cellular SnRK1 and ADK Activities Are Linked

To assess the *in vivo* relationship between SnRK1 and ADK, we quantified the activities of both kinases in extracts from transgenic plants with increased or decreased ADK or SnRK1 activity. ADK activity was measured by monitoring the production of labeled AMP following the addition of adenosine and γ^32^P-ATP to total soluble protein extracts obtained from the transgenic plants, and SnRK1 activity was determined after the addition of γ^32^P-ATP and GST-SAMS.

**Figure 7:**
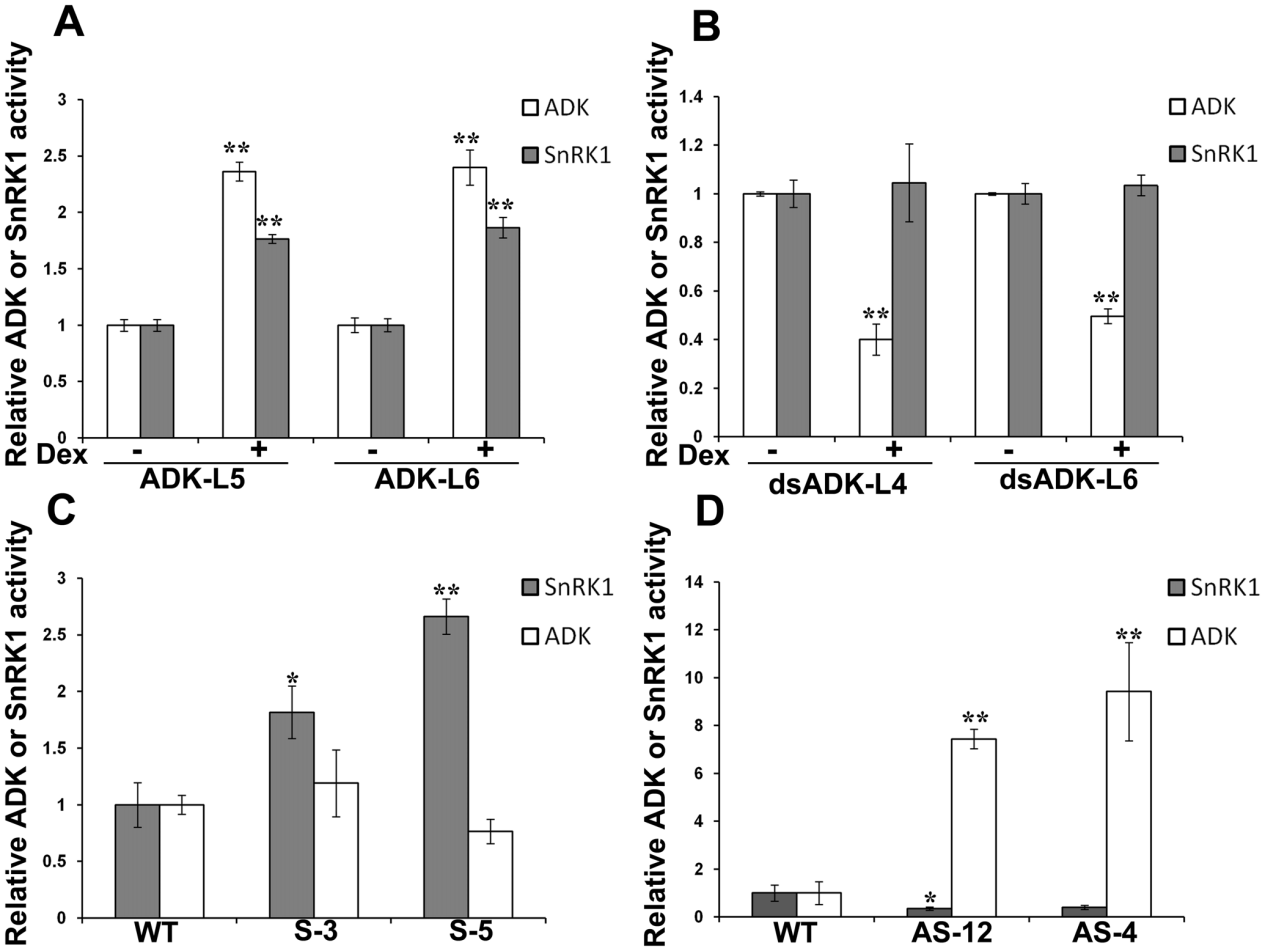
Cellular SnRK1 and ADK activities are linked. In all cases, γ^32^P-ATP and appropriate substrates were added to total soluble protein extracts to measure ADK (200 ng extract) and SnRK1 (20 µg extract) activities. (A) Arabidopsis plants containing a dexamethasone (dex)-inducible ADK transgene (lines ADK-L5 and ADK-L6) were dex- or mock-treated prior to extract preparation. (B) As before, except that ADK activity was reduced by RNA interference in Arabidopsis plants containing a dex-inducible dsADK transgene (lines dsADK-L4 and dsADK-L6). (C) Transgenic *N. benthamiana* plants (lines S-3 and S-5) expressed full-length, sense SnRK1 mRNA from the constitutive 35S promoter. Non-transgenic, wild-type (WT) plants served as control. (D) As before, except that SnRK1 activity was reduced by RNA interference in transgenic *N. benthamiana* plants (lines AS-12 and AS-4) that expressed antisense SnRK1 RNA. In (A) and (B), three independent experiments using pooled tissue from three plants, with two replicates each, were performed. In (C) and (D), two independent experiments were performed, each using two individual plants from each line and two replicate samples. Values indicate the mean +/− SE. Asterisks indicate significant differences at the 95% (*) or 99% (**) confidence level, as determined by Student's *t* test. ADK and SnRK1 activity data are presented in Tables S6A-D.

We first examined SnRK1 activity following ectopic expression of ADK. Because altering cellular ADK activity results in an extreme slow growth phenotype [36], a dexamethasone (dex)-inducible promoter was used to express this kinase [54]. Arabidopsis lines containing dex-ADK transgenes were constructed and confirmed by genomic PCR, and dex-dependent ADK expression was verified by RNA gel blot analysis and semi-quantitative RT-PCR (data not shown). Following dex or mock treatment of dex-ADK transgenic plants, extracts were obtained and tested for ADK and SnRK1 kinase activity. In the two independent lines examined (ADK-L5 and ADK-L6), it was observed that transgene expression resulted in a 2 to 2.5-fold increase in ADK activity and a 1.5 to 2-fold increase in SnRK1 activity compared to mock-treated plants (Figure 7A; Table S6A).

The effect of reducing ADK activity on endogenous SnRK1 activity was examined using previously constructed Arabidopsis lines containing a transgene that expresses double-stranded RNA corresponding to ADK2 (dsADK) from the dex promoter [40]. Because of the high degree of nucleotide sequence identity between *ADK1* and *ADK2* (88%), this construct is expected to reduce transcript levels of both genes by RNA interference. However, while considerable reductions in ADK activity were evident in the two independent lines tested (dsADK-L4 and dsADK-L6), no significant differences in SnRK1 activity were noted (Figure 7B) (Table S6B).

Reciprocal experiments employed transgenic *N. benthamiana* expressing full-length Arabidopsis SnRK1.2 (lines S-3 and S-5), or antisense SnRK1.2 RNA (lines AS-4 and AS-12) from the constitutive CaMV 35S promoter. These lines were used in an earlier study, and it was again noted that plants harboring sense transgenes exhibited a slow growth phenotype and were smaller than non-transgenic or antisense plants, which had no obvious phenotype [11]. The sense lines tested displayed a 1.5 to 2.5-fold increase in SnRK1 activity over control, non-transgenic plants. However, despite the stimulatory effect of SnRK1 observed *in vitro*, no significant alteration in ADK activity could be attributed to ectopic *in vivo* expression of SnRK1 (Figure 7C) (Table S6C). Instead, reduced SnRK1 activity mediated by the antisense SnRK1 transgenes caused dramatic increases in ADK activity (greater than 7-fold; Figure 7D) (Table S6D).

The results of these experiments show that an increase in cellular ADK activity leads to a parallel increase in SnRK1 activity, while reducing SnRK1 activity causes a substantial increase in ADK activity. Therefore, these studies demonstrate that SnRK1 and ADK activities are linked *in vivo*, but also indicate that the relationship between them is more complex than suggested by *in vitro* experiments.

## Discussion

Because SnRK1 activity is sustained by 5′-AMP, and the geminivirus AL2 and L2 pathogenicity factors interact with both SnRK1 and ADK [11], [37], we decided to study the relationship between these kinases. The experiments described here provide strong evidence that SnRK1 and ADK physically associate in a manner that is not typical of transient kinase-substrate complexes. Rather, the results of yeast two-hybrid experiments, and BiFC, co-immunoprecipitation, and co-purification studies in Arabidopsis and *N. benthamiana,* indicate that SnRK1 and ADK interact and form relatively stable cytoplasmic complexes. The results of *in vitro* studies with recombinant proteins demonstrated that ADK is phosphorylated by SnRK1-KD (albeit to a lesser extent than a GST-SAMS positive control), but also that SnRK1-KD causes a stoichiometric, rather than enzymatic, enhancement of ADK activity. That the kinase inactive SnRK1-KD-K49R also stimulates ADK confirms this and provides compelling evidence that the interaction is direct. The mechanism of non-enzymatic stimulation is not known, but could be a consequence of interaction-induced conformational changes in ADK protein. Finally, experiments with transgenic plants showed that altering the activity of either kinase affects the activity of the other, demonstrating that native SnRK1 and ADK activities are linked *in vivo*.

To maintain cellular energy charge, the SnRK1 kinase must rapidly respond to fluctuations in energy demand, suggesting that its regulation is dynamic and tightly controlled [1], [2]. The obligatory first step in SnRK1 activation is phosphorylation of the activation loop threonine, after which a key role of AMP is to prevent dephosphorylation [22], [29], [30], [31]. Interestingly, some upstream kinases are constitutively active and are not stimulated by stresses known to cause SNF1/AMPK/SnRK1 activation, suggesting an important maintenance role for AMP in kinase regulation [24], [55]. Stresses that deplete intracellular ATP result in increased AMP levels. Our data showing that ectopic expression of ADK in transgenic plants causes a corresponding increase in SnRK1 activity suggests that ADK might additionally act as an AMP source, and a SnRK1-ADK complex would be a particularly effective means of increasing local AMP concentration. SnRK1 stimulation of ADK, which we observed *in vitro*, would have the further advantage of generating a positive feedback loop for sustained response to stress. In support of the idea that ADK is a component of the stress response, ADK activity has been observed to increase following infection of *N. benthamiana* plants with DNA and RNA viruses [37].

That SnRK1 can stimulate ADK, while ADK can sustain activated SnRK1, raises the question of how the SnRK1-ADK complex might be negatively regulated. While our *in vitro* studies did not provide an answer to this question, we did observe ADK phosphorylation by SnRK1-KD, and *in vitro* and *in vivo* phosphorylation of ADK by SnRK2.8 has been reported [56]. The consequences of ADK phosphorylation *in vivo* are not known, although enzyme phosphorylation by SnRK1 is often inhibitory, if not always direct. In at least some cases, inhibition of SnRK1 targets requires subsequent binding of the phosphorylated site by one of a family of 14-3-3 proteins [7], [57]–[59]. Whether phosphorylation by SnRK1 inhibits ADK activity *in vivo* by this or some other means remains to be determined. SnRK1 activity, on the other hand, is inhibited by ATP and negatively regulated by trehalose-6-phosphate [60], [61].

While increasing cellular ADK enhanced SnRK1 activity, we did not observe a corresponding decrease when ADK activity was reduced by expression of a dsADK transgene. The absence of an effect here might be due to the fact that ADK is normally present in excess of SnRK1, or that AMP can be generated by multiple processes in the cellular environment. Somewhat surprisingly, transgenic expression of SnRK1 did not lead to increased ADK activity, as might be predicted from the results of *in vitro* experiments. However, we speculate that as yet unknown *in vivo* mechanisms (discussed above) must exist to down-regulate SnRK1-ADK complexes and note that SnRK1-expressing transgenic plants have a slow growth phenotype, indicating that an over-abundance of SnRK1 activity has adverse effects on plant physiology [11]. Thus ADK stimulation may not occur when SnRK1 activity is constitutively elevated. However, we have observed significant increases in ADK activity (greater than 5-fold) when tobacco protoplasts are metabolically stressed by treatment with 2-deoxy-D-glucose and sodium azide, both of which known to deplete cellular ATP levels and activate SnRK1 (data not shown). Conversely, the surprisingly dramatic ADK activity increase (greater than 7-fold) observed in plants with reduced SnRK1 activity due to antisense RNA expression might reflect compensatory efforts to maintain the SnRK1 activity that remains (Figure 7D). Further, if phosphorylation by SnRK1 inhibits ADK *in vivo* (as discussed above), antisense depletion of SnRK1 would be expected to result in increased ADK activity.

To put our findings in the context of a model, we speculate that cytoplasmic SnRK1-ADK complexes might facilitate the cellular response to stresses that deplete ATP. ADK generates AMP that might sustain SnRK1 activity, and SnRK1 stimulates ADK by direct physical contact, when cellular energy charge is low. ADK stimulation does not occur when ATP levels are adequate and SnRK1 activity is not required. Negative regulation may be mediated indirectly by SnRK1 phosphorylation of ADK (possibly by subsequent action of an unknown 14-3-3- protein), leading to ADK inhibition and/or disruption of SnRK1-ADK complexes. Other metabolites, such as trehalose 6-phosphate, could negatively regulate SnRK1 activity. While this model has the virtue of being simple, additional experiments will be needed to identify cellular factors involved in complex regulation. But as a crucial first step, the studies described here demonstrate that the activities of these key kinases are linked. Future work will focus on how SnRK1-ADK complexes are dynamically regulated *in vivo*, and whether ADK also enters into complexes with SNF1 and AMPK in other eukaryotes.

In conclusion, the data presented in this study provide evidence for the existence of cytoplasmic SnRK1-ADK complexes that might engender increases in local AMP concentration leading to sustained activation of SnRK1 in response to stress. We also report an apparently novel, but unknown, non-enzymatic mechanism for SnRK1-mediated stimulation of ADK that involves direct physical contact between the two kinases.

## Materials and Methods

### Protein Expression in *N. benthamiana*

SnRK1-KD, SnRK1-KD-K49R, and ADK constructs have been described [11], [37]. PCR products were cloned into PacI and AvrII digested pJL-TRBO, a TMV-based vector, to generate N-terminal, double hemagglutinin peptide-six histidine (HA_2_His_6_) fusion proteins [45], [46]. Plasmids were transformed into *Agrobacterium tumefaciens* C58C1, and cultures were used to infiltrate *N. benthamiana* leaves [38]. Tissues were collected ∼5 days post-infiltration and ground in liquid nitrogen followed by the addition of 1.25 volumes of extraction buffer (50 mM HEPES, pH 7.5, 0.1% Triton X-100, 10 mM MgCl_2_, 50 mM NaF, 1 mM EGTA, 1 mM benzamidine, 10 µM MG132-proteasomal inhibitor, 1x plant protease inhibitor cocktail (Sigma, www.sigma-aldrich.com), 5 mM β-mercaptoethanol, 0.1 mM phenylmethylsulfonyl fluoride). Extracts were filtered through miracloth and centrifuged at 12,000*g* for 20 min at 4°C. Clarified supernatant (20–25 ml) was then added to 0.5 ml of washed Nickel nitrilotriaceticacid agarose beads (Invitrogen, www.invitrogen.com) and incubated at 4°C on a rocker for 2 to 3 h. Columns were washed in 6–12 column volumes of wash buffer (50 mM NaH_2_PO_4_, pH 8.0, 300 mM NaCl, 0.1% Tween 20, 5 mM β-mercaptoethanol, with or without 20 mM imidazole). Bound HA_2_His_6_-tagged proteins were eluted with wash buffer containing 250 mM imidazole at 4°C, and then dialyzed in buffer containing 40 mM Tris-HCl, pH 7.5, and 10% glycerol. Protein concentrations were estimated using the Bradford assay (BioRad, www.bio-rad.com) with bovine serum albumin as a standard. Due to difference in the purity of purified ADK (Figure 4) and SnRK1-KD/KDKR (Figure S1), protein gel-blot signals obtained with HA antibody (the tag present in all these proteins) were used to estimate comparable protein concentrations.

### Protein Expression in *E. coli*

GST-SAMS and GST-SAMA substrates were prepared using synthetic oligonucleotides containing SAMS or SAMA peptide sequence (codons separated by periods): 5′ – CATGGGTGGT.CAT.ATG.CGT.AGC.GCG.ATG.(AGC or GCG).GGT.CTG.CAT.CTG.

GTG.AAA.CGT.CGT.GGTCAATTGGAGCT [43]. These were annealed to complementary but shorter oligonucleotides to yield duplexes with four base, single-strand overhangs (underlined). Duplex oligonucleotides were ligated into NcoI-SacI digested pGEX-KG (GE Healthcare Life Sciences, www.gelifesciences.com) containing the GST open reading frame to create C-terminal GST-SAMS and GST-SAMA fusion proteins. Proteins were expressed in *E. coli* strain BL21 (DE3) and purified by glutathione-agarose chromatography. GST-SnRK1-KD and GST-SnRK1-KD-K49R proteins were similarly prepared, expressed, and purified [11]. Protein concentrations were estimated using the Bradford assay (BioRad, www.bio-rad.com) with bovine serum albumin as a standard.

### Kinase Assays

SnRK1-KD autophosphorylation, and GST-SAMS and ADK phosphorylation (by SnRK1-KD) assays were performed as described [62]. Reactions contained 10 to 30 ng of SnRK1-KD (or SnRK1-KD K49R) with or without 3 µg of substrate proteins (ADK or GST-SAMS). Reactions with partially purified proteins were initiated by addition of 0.5 mM unlabeled ATP and incubated at 30°C for 20 min to activate SnRK1-KD, and also in some cases to obscure autophosphorylation. Unlabeled ATP was removed from SnRK1-KD preparations using Zeba Spin desalting columns (Thermo Scientific, www.thermoscientific.com/pierce). γ^32^P-ATP (3000 Ci/mmol; Perkin Elmer, www.perkinelmer.com) was then added to a final concentration of 0.05 µM along with substrate protein, and the reaction mixture incubated a further 30 min at 30°C before electrophoresis on 10% polyacrylamide-SDS gels. In autophosphorylation assays and experiments involving immunoprecipitates or cell extracts, pre-incubation with unlabeled ATP was usually excluded. Labeled proteins were visualized and quantitated using a phosphor-imager (BioRad, www.bio-rad.com). ADK assays were performed with γ^32^P-ATP and employed thin layer chromatography to separate the adenosine substrate from labeled AMP product [37].

### Enzyme Activity Measurements and Statistical Analysis

For SnRK1 activity measurements, the intensity of ^32^P-labeled GST-SAMS or autophosphorylation signal was determined using Quantity one software (Bio-Rad) from images obtained by exposing assay gels to a phosphor-imager screen.

For ADK activity measurements, the intensity of ^32^P-labeled AMP was determined similarly, using Quantity one software (Bio-Rad) from images obtained by exposing the TLC assay plates to a phosphor-imager screen. In all ADK assays, signal obtained in the absence of enzyme was subtracted from readings obtained in the presence of enzyme. In experiments measuring ADK activity in the presence of various proteins (Figure 6; Table S4A-E), ADK activities were normalized to those obtained in the presence of GFP (for plant-expressed HA_2_His_6_-fusion proteins) or GST (for *E. coli*-expressed GST-fusion proteins).

In transgenic plants under- or overexpressing ADK (*A. thaliana*) or SnRK1 (*N. benthamiana*), relative ADK and SnRK1 activities were normalized to control group plants (mock-induced transgenic *A. thaliana* transgenic plants or wild type *N. benthamiana* plants; Figures 6 and 7).

Data obtained in all the above experiments were subjected to Students t-test using the Excel program (Microsoft Office-2003) with one-tailed distribution and two-sample with equal variance (homoscedastic) settings, to test if differences in relative SnRK1 and ADK activities between experimental and control groups were statistically significant. The same software program was used to calculate standard error (SE) of the mean.

### Protein Interaction Analysis

The yeast two-hybrid system was used to identify interactions between ADK and SnRK1 proteins [47], [63]. Bait and prey constructs containing ADK, SnRK1, and SnRK1-KD, and analysis methods, have been described [11], [37].

BiFC vectors containing the N- or C-terminal portions of enhanced yellow fluorescent protein (YFP) for fusion to the N- or C-terminus of test proteins (pYN and pYC, or p2YN and p2YC, respectively) have been described [48], [49], as have plasmids containing Arabidopsis ADK2 and SnRK1.2 [11], [37]. ADK2 was amplified by PCR using forward primer 5′-CCCTTAATTAACATG.GCT.TCT.TCT.TCT.AAC.TAC and reverse primer 5′-GGGACTAGT.GTT.AAA.GTC.GGG.TTT.CTC.AGG. SnRK1.2 was amplified with forward primer 5′-CCCTTAATTAACATG.GAT.CAT.TCA.TCA.AAT.AG and reverse primer 5′-GGGACTAGT.GAT.CAC.ACG.AAG.CTC.TGT.AAG (periods separate codons and PacI and SpeI sites are underlined). PCR products were digested with these enzymes and ligated into similarly cleaved BiFC vectors. Plasmids were transformed into *A. tumefaciens* C58C1, and cultures were used to infiltrate *N. benthamiana* leaves [49]. Cultures containing YN- and YC-based plasmids were mixed 1∶1 immediately prior to infiltration. Histone 2b fused to red fluorescent protein (RFP-H2B) was used as a nuclear marker [64]. Leaf tissue was analyzed by microscopy ∼48 h post-infiltration using a Nikon PCM 2000 (Nikon, www.nikon.com) confocal laser scanning microscope. To record YFP fluorescence, a band-pass emission filter (EM515/30HQ) with a 450- to 490 nm excitation wavelength and 515 nm emission wavelength was used. To record RFP fluorescence, a 565 nm long-pass filter (E565LP) was employed. Images were captured using Simple PCI software and compiled with Adobe Photoshop (Adobe, www.adobe.com).

### Immunoprecipitation and Immunodetection

Immunoprecipitation with anti-ADK antibody and anti-TMD1-antibody was performed using crude extracts from 4 week-old Arabidopsis (Col-0) plants. Immunoprecipitates were subjected to gel blot analysis using antibodies against ADK [37], Human AMPK pT172 antibody to detect SnRK1 (Santa Cruz Biotechnology, www.scbt.com), and TMD1.

### Transgenic Plant Studies

Construction of transgenic Arabidopsis containing a dex-inducible dsADK construct, and methods for dex induction, have been described [40]. Similar procedures were used to generate and analyze transgenic lines expressing ADK from the dex promoter. Transgenic *N. benthamiana* lines expressing SnRK1.2 or antisense SnRK1.2 mRNA have been described [11].

## Supporting Information

Figure S1 **Partially purified SnRK1-KD and SnRK1-KD-K49R proteins.** Coomassie blue-stained SDS-PAGE gel images are shown. (A) HA_2_His_6_-SnRK1-KD and HA_2_His_6_-SnRK1-KD-K49R (∼42 kDa) expressed and purified from *N. benthamiana*. (B) GST-SnRK1-KD and GST-SnRK1-KD-K49R (∼68 kDa) expressed and purified from *E. coli*. Protein identities were verified by immunoblots with anti-HA or anti-GST probes, as appropriate.
(TIF)

Figure S2 **SnRK1-KD expressed in** ***E. coli*** **has basal activity, while SnRK1-KD expressed in** ***N. benthamiana*** **is highly active.** A comparison of kinase activities following expression and purification from *E. coli* (GST-SnRK1-KD) or *N. benthamiana* (HA_2_His_6_-SnRK1-KD) is presented. Kinase inactive mutant proteins (SnRK1-KD-K49R) were employed as negative controls. The reactions shown contained the indicated proteins and γ^32^P-ATP. Following kinase reactions, proteins were subjected to SDS-PAGE and exposed to a phosphor-imager for 5 h to detect labeled proteins. Asterisks (*) indicate lanes that were exposed for 72 h to detect GST-SnRK1-KD activity. Immunoblots using anti-HA (α-HA) and anti-GST (α-GST) were also used to detect proteins. (A) SnRK1-KD autophosphorylation. Activity was tested in the absence of added substrate. (B) SnRK1-KD phosphorylation of GST-SAMS. Activity was tested using GST-SAMS substrate, or GST-SAMA as a negative control substrate. (C) SnRK1-KD phosphorylation of ADK. Activity was tested using ADK as substrate. To obscure autophosphorylation, HA_2_His_6_-SnRK1-KD and GST-SnRK1-KD were pre-incubated with 0.5 mM unlabeled ATP for 20 min in kinase buffer before adding ADK and γ^32^P-ATP.
(TIF)

Figure S3 **5′-AMP or ADK do not stimulate SnRK1-KD activity** ***in vitro*** **.** HA_2_His_6_-SnRK1-KD autophosphorylation activity was assessed alone or in the presence of a two-fold molar excess of ADK protein, or varying amounts of 5′-AMP as indicated. Following kinase reactions, which included γ^32^P-ATP, samples were fractionated on SDS-PAGE and exposed to a phosphor-imager to detect labeled protein.
(TIF)

Table S1 **Activity of SnRK1-KD expressed in** ***N. benthamiana*** **.** Activity values (in arbitrary units) were obtained by measuring signal intensity of ^32^P-labeled SnRK1-KD or SnRK1-KD-K49R (autophosphorylation), or GST-SAMS or GST-SAMA, from images obtained by exposing the PAGE gels to a phosphor-imager (Figure 1B and 1C).
(PDF)

Table S2 **ADK proteins from diverse eukaryotes contain conserved potential SNF1/AMPK/SnRK1 phosphorylation sites.** ADK protein sequences were compared to determine the extent of conservation at sites corresponding to T68, T105, S175, and S196 in *Arabidopsis* ADK1 and ADK2. Amino acids shown in red are important for SNF1/AMPK/SnRK1 recognition, and serine/threonine residues underlined are predicted phosphorylation sites. A hyphen (-) indicates the site is not conserved.
(PDF)

Table S3 **Phosphorylation of ADK and GST-SAMS by SnRK1-KD expressed in** ***N. benthamiana*** **.** Activity values (in arbitrary units) were obtained by measuring signal intensity of ^32^P-labeled SnRK1-KD (10 ng) or SnRK1-KD-K49R (30 ng) from PAGE gels exposed to a phosphor-imager (autophosphorylation, Figure 5A). Activity values are also included for SnRK1-KD pre-incubated with cold ATP (autophosphorylation), and pre-incubated SnRK1-KD (10 ng) or SnRK1-KD-K49R (30 ng) +ADK in the presence of ^32^P-ATP (Figure 5B). Images used for GST-SAMS incubated with the same SnRK1-KD (10 ng) or SnRK-KD-K49R (30 ng) preparations and ^32^P-ATP are shown in Figure 1C.
(PDF)

Table S4 **A. ADK activity in the presence of HA_2_-His_6_-SnRK1-KD/K49R expressed in** ***N.*** ** ***benthamiana*** **.** Activity of purified ADK was measured in the presence of HA_2_His_6_-SnRK1-KD, HA_2_His_6_-SnRK1-KDK49R (kinase inactive mutant), and additional control proteins expressed and partially purified from *N. benthamiana*. ADK activity values (in arbitrary units) were obtained by measuring signal intensity of ^32^P-labeled reaction product (5′-AMP) from TLC plates exposed to a phosphor-imager. Data were obtained from three independent experiments with two replicates each, and are shown graphically in Figure 6A. **B. ADK activity in the presence of GST-SnK1-KD/K49R expressed in** ***E. coli*** **.** Activity of purified ADK was measured in the presence of GST-SnRK1-KD or GST-SnRK1-KDK49R (kinase inactive mutant) expressed and partially purified from *E. coli*. ADK activity values (in arbitrary units) were obtained by measuring signal intensity of ^32^P-labeled reaction product (5′-AMP) from TLC plates exposed to a phosphor-imager. Data were obtained from three independent experiments with two replicates each, and are shown graphically in Figure 6B. **C. Stimulation of ADK activity by SnRK1-KD.** Activity of purified ADK was measured in the presence of HA_2_His_6_-SnRK1-KD expressed in *N. benthamiana*, or GST-SnRK1-KD expressed in *E. coli*. ADK activity values (in arbitrary units) were obtained by measuring signal intensity of ^32^P-labeled reaction product (5′-AMP) from TLC plates exposed to a phosphor-imager. Data were obtained from three independent experiments, and are shown graphically in Figure 6C. *For fold-change calculations, ADK activities were normalized to ADK+GFP or ADK+GST, as appropriate.
(PDF)

Table S5 **Activity of SnRK1-KD expressed in** ***E. coli*** **and** ***N. benthamiana*** **.** HA_2_His_6_-SnRK1-KD was expressed and partially purified from *N. benthamiana*, while GST-SnRK1-KD* was expressed and partially purified from *E. coli*. Activity values (arbitrary units) were obtained by measuring signal intensity of ^32^P-labeled SnRK1-KD (autophosphorylation) or GST-SAMS from images obtained by exposing PAGE gels to a phosphor-imager (Figure S2). Exposures were for 5 h in the case of HA_2_His_6_-SnRK1-KD, and 72 h for GST-SnRK1-KD*.
(PDF)

Table S6 **A. ADK and SnRK1 activities in ADK over-expression lines.** Activities of ADK and SnRK1 in crude extracts from transgenic Arabidopsis lines ADK-L5 and ADK-L6 expressing ADK from a dexamethasone (dex) inducible promoter are shown. Data were obtained from three independent experiments using pooled tissue from three plants, with two replicates each. Activity values (arbitrary units) were obtained by measuring signal intensity of ^32^P labeled GST-SAMS (SnRK1 substrate) or 5′AMP (ADK product) by exposing PAGE gels or TLC plates, respectively, to a phosphor-imager. Data are shown graphically in Figure 7A. **B. ADK and SnRK1 activities in ADK RNAi lines.** Activities of ADK and SnRK1 in crude extracts from transgenic Arabidopsis lines dsADK-L4 and dsADK-L6 expressing ADK dsRNA from a dexamethasone (dex) inducible promoter are shown. Data were obtained from three independent experiments using pooled tissue from three plants, with two replicates each. Activity values (arbitrary units) were obtained by measuring signal intensity of ^32^P labeled GST-SAMS (SnRK1 substrate) or 5′AMP (ADK product) by exposing PAGE gels or TLC plates, respectively, to a phosphor-imager. Data are shown graphically in Figure 7B. **C. SnRK1 and ADK activities in SnRK1 over-expression lines.** SnRK1 and ADK activities in crude extracts from transgenic *N. benthamiana* lines S3 and S5 expressing SnRK1 from the constitutive 35S promoter are shown. Data were obtained from two independent experiments using two individual plants from each line and two replicate samples. Activity values (arbitrary units) were obtained by measuring signal intensity of ^32^P labeled GST-SAMS (SnRK1 substrate) or 5′AMP (ADK product) by exposing PAGE gels or TLC plates, respectively, to a phosphor-imager. Data are shown graphically in Figure 7C. **D. SnRK1 and ADK activities in SnRK1 antisense lines.** SnRK1 and ADK activities in crude extracts from transgenic *N. benthamiana* lines AS-4 and AS-12 expressing antisense SnRK1 RNA from the constitutive 35S promoter are shown. Data were obtained from two independent experiments using two individual plants from each line and two replicate samples. Activity values (arbitrary units) were obtained by measuring signal intensity of ^32^P labeled GST-SAMS (SnRK1 substrate) or 5′AMP (ADK product) by exposing PAGE gels or TLC plates, respectively, to a phosphor-imager. Data are shown graphically in Figure 7D.
(PDF)
