# Supplementary figures and images for: A Complex Containing SNF1-Related Kinase (SnRK1) and Adenosine Kinase in Arabidopsis
Source: PLoS One. 2014 Jan 30;9(1):e87592. doi: 10.1371/journal.pone.0087592 (PMC3907550; doi:10.1371/journal.pone.0087592)

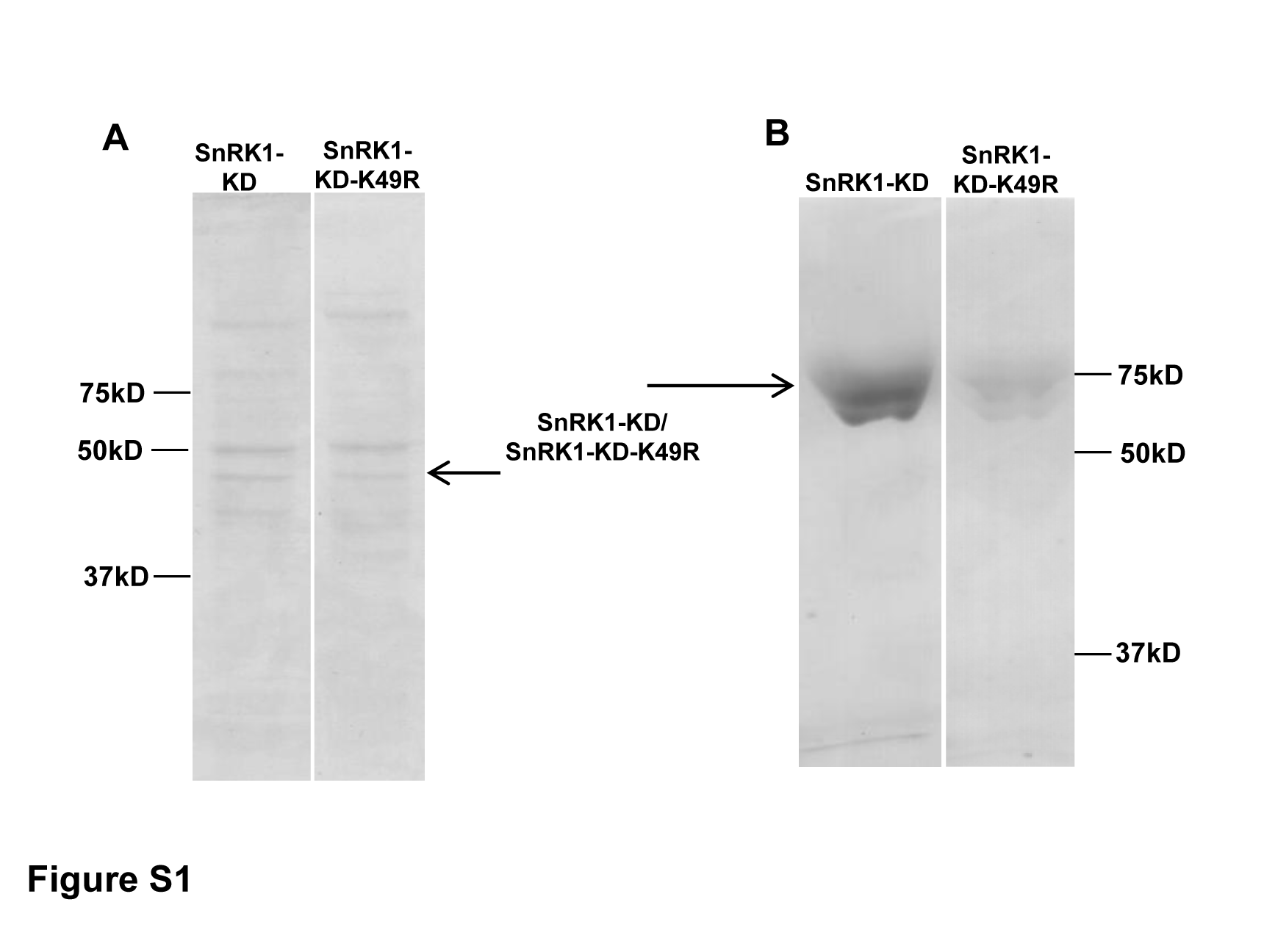

Supplement: Figure S1 — Partially purified SnRK1-KD and SnRK1-KD-K49R proteins. Coomassie blue-stained SDS-PAGE gel images are shown. (A) HA2His6-SnRK1-KD and HA2His6-SnRK1-KD-K49R (∼42 kDa) expressed and purified from N. benthamiana. (B) GST-SnRK1-KD and GST-SnRK1-KD-K49R (∼68 kDa) expressed and purified from E. coli. Protein identities were verified by immunoblots with anti-HA or anti-GST probes, as appropriate. (TIF) [file pone.0087592.s001.tif]

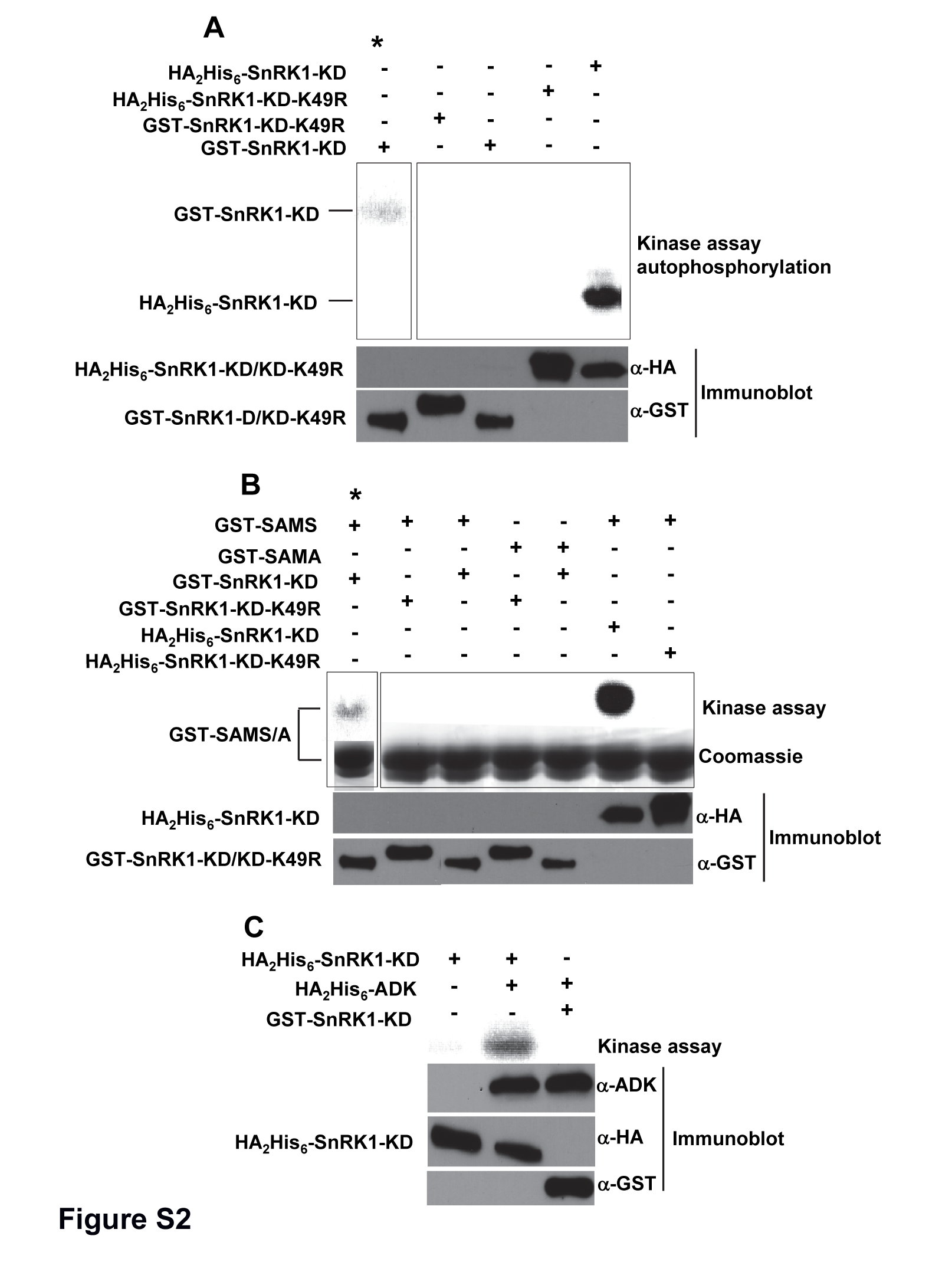

Supplement: Figure S2 — SnRK1-KD expressed in E. coli has basal activity, while SnRK1-KD expressed in N. benthamiana is highly active. A comparison of kinase activities following expression and purification from E. coli (GST-SnRK1-KD) or N. benthamiana (HA2His6-SnRK1-KD) is presented. Kinase inactive mutant proteins (SnRK1-KD-K49R) were employed as negative controls. The reactions shown contained the indicated proteins and γ32P-ATP. Following kinase reactions, proteins were subjected to SDS-PAGE and exposed to a phosphor-imager for 5 h to detect labeled proteins. Asterisks (*) indicate lanes that were exposed for 72 h to detect GST-SnRK1-KD activity. Immunoblots using anti-HA (α-HA) and anti-GST (α-GST) were also used to detect proteins. (A) SnRK1-KD autophosphorylation. Activity was tested in the absence of added substrate. (B) SnRK1-KD phosphorylation of GST-SAMS. Activity was tested using GST-SAMS substrate, or GST-SAMA as a negative control substrate. (C) SnRK1-KD phosphorylation of ADK. Activity was tested using ADK as substrate. To obscure autophosphorylation, HA2His6-SnRK1-KD and GST-SnRK1-KD were pre-incubated with 0.5 mM unlabeled ATP for 20 min in kinase buffer before adding ADK and γ32P-ATP. (TIF) [file pone.0087592.s002.tif]

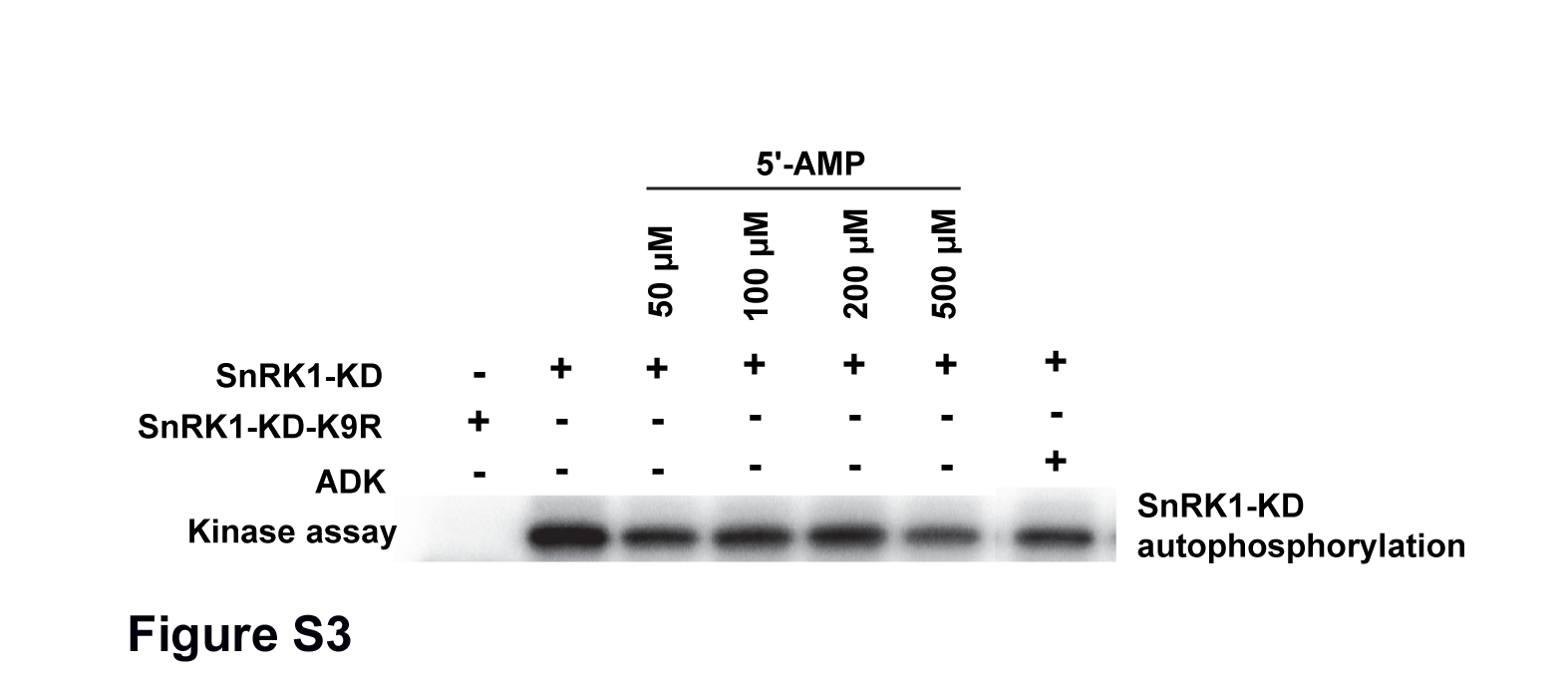

Supplement: Figure S3 — 5′-AMP or ADK do not stimulate SnRK1-KD activity in vitro . HA2His6-SnRK1-KD autophosphorylation activity was assessed alone or in the presence of a two-fold molar excess of ADK protein, or varying amounts of 5′-AMP as indicated. Following kinase reactions, which included γ32P-ATP, samples were fractionated on SDS-PAGE and exposed to a phosphor-imager to detect labeled protein. (TIF) [file pone.0087592.s003.tif]
