# Supplementary material for: A Complex Containing SNF1-Related Kinase (SnRK1) and Adenosine Kinase in Arabidopsis
Source: PLoS One. 2014 Jan 30;9(1):e87592. doi: 10.1371/journal.pone.0087592 (PMC3907550; doi:10.1371/journal.pone.0087592)
Supplement: Table S1 — Activity of SnRK1-KD expressed in N. benthamiana . Activity values (in arbitrary units) were obtained by measuring signal intensity of 32P-labeled SnRK1-KD or SnRK1-KD-K49R (autophosphorylation), or GST-SAMS or GST-SAMA, from images obtained by exposing the PAGE gels to a phosphor-imager (Figure 1B and 1C). (PDF) [file pone.0087592.s004.pdf]

Table S1. Activity of SnRK1-KD expressed in *N. benthamiana*

SnRK1-KD or SnRK1-KD-K49R* (ng)	Substrate	SnRK1 activity
5	GST-SAMS	145122
10	GST-SAMS	261765
15	GST-SAMS	295594
20	GST-SAMS	414018
20	GST-SAMA	5377
30*	GST-SAMS	15157
30*	GST-SAMA	2931
20	auto	248345
30*	auto	11928

Activity values (in arbitrary units) were obtained by measuring signal intensity of ^{32}P-labeled SnRK1-KD or SnRK1-KD-K49R (autophosphorylation), or GST-SAMS or GST-SAMA, from images obtained by exposing PAGE gels to a phosphorimager (Figure 1B and 1C).
