# Supplementary material for: A Complex Containing SNF1-Related Kinase (SnRK1) and Adenosine Kinase in Arabidopsis
Source: PLoS One. 2014 Jan 30;9(1):e87592. doi: 10.1371/journal.pone.0087592 (PMC3907550; doi:10.1371/journal.pone.0087592)
Supplement: Table S2 — ADK proteins from diverse eukaryotes contain conserved potential SNF1/AMPK/SnRK1 phosphorylation sites. ADK protein sequences were compared to determine the extent of conservation at sites corresponding to T68, T105, S175, and S196 in Arabidopsis ADK1 and ADK2. Amino acids shown in red are important for SNF1/AMPK/SnRK1 recognition, and serine/threonine residues underlined are predicted phosphorylation sites. A hyphen (-) indicates the site is not conserved. (PDF) [file pone.0087592.s005.pdf]

Table S2. ADK proteins from diverse eukaryotes contain conserved potential SNF1/AMPK/SnRK1 phosphorylation sites

Species	% Identity, entire protein	T68	T105	S175	S196
Arabidopsis thaliana (ADK2)	100	IAGGATQNSI	MKKDATAAGV	FFLTVSPESI	FTMNLSAPFI
Arabidopsis thaliana (ADK1)	92	IAGGATQNSI	MKKDATAAGV	FFLTVSPESI	FTMNLSAPFI
Vitis vinifera	86	IAGGATQNSI	-	FFLTVSPESI	FMMNLSAPFI
Populus trichocarpa	86	IAGGATQNSI	MKKNSTEAGV	FFLTVSPESI	FTMNLSAPFI
Solanum tuberosum	85	IAGGATQNSI	-	FFLTVSPESI	FSMNLSAPFI
Nicotiana tabacum	86	IAGGATQNSI	-	FFLTVSPESI	FSMNLSAPFI
Gossypium hirsutum	85	IAGGATQNSI	-	FFLTVSPESI	FSMNLSAPFI
Sorghum bicolor	82	IAGGATQNSI	-	FFLTVSPDSI	FMMNLSAPFI
Oryza sativa	82	IAGGATQNSI	-	FFLTVSPDSI	FLMNLSAPFI
Glycine max	81	IAGGATQNSI	-	FFLTVSPDSI	FSMNLSAPFI
Zea mays	82	IAGGATQNSI	-	FFLTVSPDSI	FLMNLSAPFI
Physcomitrella patens	67	IAGGATQNTI	MFKLASEGGV	FFLTVSPESM	FMMNLSASFV
Chlamydomonas reinhardtii	53	-	MTEVATAEGV	FFITVSPASI	-
Mus musculus	57	-	-	FFLTVSPESV	FTLNLSAPFI
Danio rerio	59	-	-	FFLTVSLESI	FCLNLSAPFI
Rattus norvegicus	57	-	-	FTMNLSAPFV	FTLNLSAPFI
Macaca mulatta	56	-	-	FFLTVSPESV	FTLNLSAPFI
Homo sapiens	56	-	-	FFLTVSPESV	FTLNLSAPFI
Saccharomyces cerevisiae	40	-	-	FHLTVSPDAI	FVLNFSAPFI

ADK proteins from diverse eukaryotes contain conserved potential SnRK1 phosphorylation sites. ADK protein sequences were compared to determine the extent of conservation at sites corresponding to T68, T105, S175, and S196 in *Arabidopsis* ADK1 and ADK2. Amino acids shown in red are critical for SnRK1 recognition, and serine/threonine residues underlined are predicted to be phosphorylated by SnRK1. A hyphen (-) indicates the site is not conserved.
