# Supplementary material for: A Complex Containing SNF1-Related Kinase (SnRK1) and Adenosine Kinase in Arabidopsis
Source: PLoS One. 2014 Jan 30;9(1):e87592. doi: 10.1371/journal.pone.0087592 (PMC3907550; doi:10.1371/journal.pone.0087592)
Supplement: Table S3 — Phosphorylation of ADK and GST-SAMS by SnRK1-KD expressed in N. benthamiana . Activity values (in arbitrary units) were obtained by measuring signal intensity of 32P-labeled SnRK1-KD (10 ng) or SnRK1-KD-K49R (30 ng) from PAGE gels exposed to a phosphor-imager (autophosphorylation, Figure 5A). Activity values are also included for SnRK1-KD pre-incubated with cold ATP (autophosphorylation), and pre-incubated SnRK1-KD (10 ng) or SnRK1-KD-K49R (30 ng) +ADK in the presence of 32P-ATP (Figure 5B). Images used for GST-SAMS incubated with the same SnRK1-KD (10 ng) or SnRK-KD-K49R (30 ng) preparations and 32P-ATP are shown in Figure 1C. (PDF) [file pone.0087592.s006.pdf]

Table S3. Phosphorylation of ADK and GST-SAMS by SnRK1-KD expressed in *N. benthamiana*

SnRK1-KD or SnRK1-KD-K49R* (ng)	Substrate	SnRK1 activity
10	auto†	235904
30*	auto†	23421
10	auto	22631
10	ADK	84421
30*	ADK	34076
10	GST-SAMS	261765
30*	GST-SAMS	15157

† Not pre-incubated with unlabeled ATP

Activity values (in arbitrary units) were obtained by measuring signal intensity of ^{32}P-labeled SnRK1-KD (10 ng) or SnRK1-KD-K49R (30 ng) from PAGE gels exposed to a phosphorimager (autophosphorylation, Figure 5A). Activity values are also included for SnRK1-KD pre-incubated with cold ATP (autophosphorylation), and pre-incubated SnRK1-KD (10 ng) or SnRK1-KD-K49R (30 ng) + ADK in the presence of ^{32}P-ATP (Figure 5B). Images used for GST-SAMS incubated with the same SnRK1-KD (10 ng) or SnRK-KD-K49R (30 ng) preparations and ^{32}P-ATP are shown in Figure 1C.
