# Supplementary material for: A Complex Containing SNF1-Related Kinase (SnRK1) and Adenosine Kinase in Arabidopsis
Source: PLoS One. 2014 Jan 30;9(1):e87592. doi: 10.1371/journal.pone.0087592 (PMC3907550; doi:10.1371/journal.pone.0087592)
Supplement: Table S4 — A. ADK activity in the presence of HA2-His6-SnRK1-KD/K49R expressed in N. benthamiana . Activity of purified ADK was measured in the presence of HA2His6-SnRK1-KD, HA2His6-SnRK1-KDK49R (kinase inactive mutant), and additional control proteins expressed and partially purified from N. benthamiana. ADK activity values (in arbitrary units) were obtained by measuring signal intensity of 32P-labeled reaction product (5′-AMP) from TLC plates exposed to a phosphor-imager. Data were obtained from three independent experiments with two replicates each, and are shown graphically in Figure 6A. B. ADK activity in the presence of GST-SnK1-KD/K49R expressed in E. coli . Activity of purified ADK was measured in the presence of GST-SnRK1-KD or GST-SnRK1-KDK49R (kinase inactive mutant) expressed and partially purified from E. coli. ADK activity values (in arbitrary units) were obtained by measuring signal intensity of 32P-labeled reaction product (5′-AMP) from TLC plates exposed to a phosphor-imager. Data were obtained from three independent experiments with two replicates each, and are shown graphically in Figure 6B. C. Stimulation of ADK activity by SnRK1-KD. Activity of purified ADK was measured in the presence of HA2His6-SnRK1-KD expressed in N. benthamiana, or GST-SnRK1-KD expressed in E. coli. ADK activity values (in arbitrary units) were obtained by measuring signal intensity of 32P-labeled reaction product (5′-AMP) from TLC plates exposed to a phosphor-imager. Data were obtained from three independent experiments, and are shown graphically in Figure 6C. *For fold-change calculations, ADK activities were normalized to ADK+GFP or ADK+GST, as appropriate. (PDF) [file pone.0087592.s007.pdf]

Table S.4A. ADK activity in the presence of HA_2-His_6-SnRK1-KD/K49R expressed in *N. benthamiana*

	ADK activity	Fold change over (ADK+GFP) Mean±SE	Students t-test values relative to ADK+GFP	Students t-test values relative to ADK+SnRK1-KD
ADK	154461	0.480±0.050	0.0157	-
ADK+GFP	329790	1.000±0.109	-	-
ADK+APT1	264074	0.800±0.049	0.2258	-
ADK+SnRK1-KD	2313884	6.917±0.255	0.0013	-
ADK+SnRK1-KDK49R	1906351	5.667±0.308	0.0012	0.265

Activity of purified ADK was measured in the presence of HA_2His_6-SnRK1-KD, HA_2His_6-SnRK1-KDK49R (kinase inactive mutant), and additional control proteins expressed and partially purified from *N. benthamiana*. ADK activity values (in arbitrary units) were obtained by measuring signal intensity of ^{32}P-labeled reaction product (5'-AMP) from TLC plates exposed to a phosphor-imager. Data were obtained from three independent experiments with two replicates each, and are shown graphically in Figure 6A.

Table S4B. ADK activity in the presence of GST-SnK1-KD/K49R expressed in *E. coli*

	ADK activity	Fold change over (ADK+GST) Mean±SE	Students t-test values relative to ADK+GST	Students t-test values relative to ADK+SnRK1-KD
ADK	350663	0.272±0.012	6.07E-04	-
ADK+GST	1273509	1.000±0.088	-	-
ADK+SnRK1-KD	5936427	4.795±0.205	1.47E-05	-
ADK+SnRK1-KD-K49R	5494783	4.418±0.159	1.15E-04	0.327

Activity of purified ADK was measured in the presence of GST-SnRK1-KD or GST-SnRK1-KDK49R (kinase inactive mutant) expressed and partially purified from *E. coli*. ADK activity values (in arbitrary units) were obtained by measuring signal intensity of ^{32}P-labeled reaction product (5'-AMP) from TLC plates exposed to a phosphor-imager. Data were obtained from three independent experiments with two replicates each, and are shown graphically in Figure 6B.

Table S4C. Stimulation of ADK activity by SnRK1-KD

SnRK1-ADK ratio	ADK activity in the presence of: HA_2His_6-SnRK1-KD	GST-SnRK1-KD	*Fold change in ADK activity: HA_2His_6-SnRK1-KD	GST-SnRK1-KD
0.00	663209	526345	0.55	0.37
0.02	989658	727428	0.82	0.51
0.10	2021005	1872595	1.68	1.32
0.20	3988985	4057378	3.32	2.86
0.60	8443371	5063884	7.03	3.57
1.50	10017662	6680336	8.34	4.71
4.00	9051365	6379880	7.53	4.50

Activity of purified ADK was measured in the presence of HA_2His_6-SnRK1-KD expressed in *N. benthamiana*, or GST-SnRK1-KD expressed in *E. coli*. ADK activity values (in arbitrary units) were obtained by measuring signal intensity of ^{32}P-labeled reaction product (5'-AMP) from TLC plates exposed to a phosphor-imager. Data were obtained from three independent experiments, and are shown graphically in Figure 6C.

*For fold-change calculations, ADK activities were normalized to ADK + GFP or ADK + GST, as appropriate.
