# Supplementary material for: A Complex Containing SNF1-Related Kinase (SnRK1) and Adenosine Kinase in Arabidopsis
Source: PLoS One. 2014 Jan 30;9(1):e87592. doi: 10.1371/journal.pone.0087592 (PMC3907550; doi:10.1371/journal.pone.0087592)
Supplement: Table S5 — Activity of SnRK1-KD expressed in E. coli and N. benthamiana . HA2His6-SnRK1-KD was expressed and partially purified from N. benthamiana, while GST-SnRK1-KD* was expressed and partially purified from E. coli. Activity values (arbitrary units) were obtained by measuring signal intensity of 32P-labeled SnRK1-KD (autophosphorylation) or GST-SAMS from images obtained by exposing PAGE gels to a phosphor-imager (Figure S2). Exposures were for 5 h in the case of HA2His6-SnRK1-KD, and 72 h for GST-SnRK1-KD*. (PDF) [file pone.0087592.s008.pdf]

**Table S5. Activity of SnRK1-KD expressed in *E. coli* and *N. benthamiana***

| | Substrate | SnRK1 activity |
|---|---|---|
| GST-SnRK1-KD* | auto | 2290 |
| $HA_2His_6$-SnRK1-KD | auto | 750709 |
| GST-SnRK1-KD* | GST-SAMS | 4211 |
| $HA_2His_6$-SnRK1-KD | GST-SAMS | 1022329 |

$HA_2His_6$-SnRK1-KD was expressed and partially purified from *N. benthamiana*, while GST-SnRK1-KD* was expressed and partially purified from *E. coli*. Activity values (arbitrary units) were obtained by measuring signal intensity of $^{32}P$-labeled SnRK1-KD (autophosphorylation) or GST-SAMS from images obtained by exposing PAGE gels to a phosphorimager (Figure S2). Exposures were for 5 h in the case of $HA_2His_6$-SnRK1-KD, and 72 h for GST-SnRK1-KD*.
