# Supplementary material for: A Complex Containing SNF1-Related Kinase (SnRK1) and Adenosine Kinase in Arabidopsis
Source: PLoS One. 2014 Jan 30;9(1):e87592. doi: 10.1371/journal.pone.0087592 (PMC3907550; doi:10.1371/journal.pone.0087592)
Supplement: Table S6 — A. ADK and SnRK1 activities in ADK over-expression lines. Activities of ADK and SnRK1 in crude extracts from transgenic Arabidopsis lines ADK-L5 and ADK-L6 expressing ADK from a dexamethasone (dex) inducible promoter are shown. Data were obtained from three independent experiments using pooled tissue from three plants, with two replicates each. Activity values (arbitrary units) were obtained by measuring signal intensity of 32P labeled GST-SAMS (SnRK1 substrate) or 5′AMP (ADK product) by exposing PAGE gels or TLC plates, respectively, to a phosphor-imager. Data are shown graphically in Figure 7A. B. ADK and SnRK1 activities in ADK RNAi lines. Activities of ADK and SnRK1 in crude extracts from transgenic Arabidopsis lines dsADK-L4 and dsADK-L6 expressing ADK dsRNA from a dexamethasone (dex) inducible promoter are shown. Data were obtained from three independent experiments using pooled tissue from three plants, with two replicates each. Activity values (arbitrary units) were obtained by measuring signal intensity of 32P labeled GST-SAMS (SnRK1 substrate) or 5′AMP (ADK product) by exposing PAGE gels or TLC plates, respectively, to a phosphor-imager. Data are shown graphically in Figure 7B. C. SnRK1 and ADK activities in SnRK1 over-expression lines. SnRK1 and ADK activities in crude extracts from transgenic N. benthamiana lines S3 and S5 expressing SnRK1 from the constitutive 35S promoter are shown. Data were obtained from two independent experiments using two individual plants from each line and two replicate samples. Activity values (arbitrary units) were obtained by measuring signal intensity of 32P labeled GST-SAMS (SnRK1 substrate) or 5′AMP (ADK product) by exposing PAGE gels or TLC plates, respectively, to a phosphor-imager. Data are shown graphically in Figure 7C. D. SnRK1 and ADK activities in SnRK1 antisense lines. SnRK1 and ADK activities in crude extracts from transgenic N. benthamiana lines AS-4 and AS-12 expressing antisense SnRK1 RNA from the constitutive [file pone.0087592.s009.pdf]

Table S6A. ADK and SnRK1 activities in ADK over-expression lines

	ADK activity			SnRK1 activity			t test values	t test values
	Mock	Dex	Fold change (Dex/Mock) Mean±SE	Mock	Dex	Fold change (Dex/Mock) Mean±SE	ADK activity	SnRK1 activity
ADK-L5	294730	690202	2.363±0.083	32974	58334	1.765±0.039	3.10E-09	4.90E-05
ADK-L6	438596	1069406	2.397±0.156	45305	83349	1.865±0.091	6.10E-04	7.05E-08

Activities of ADK and SnRK1 in crude extracts from transgenic Arabidopsis lines ADK-L5 and ADK-L6 expressing ADK from a dexamethasone (dex) inducible promoter are shown. Data were obtained from three independent experiments using pooled tissue from three plants, with two replicates each. Activity values (arbitrary units) were obtained by measuring signal intensity of ^{32}P labeled GST-SAMS (SnRK1 substrate) or 5'AMP (ADK product) by exposing PAGE gels or TLC plates, respectively, to a phosphorimager. Data are shown graphically in Figure 7A.

Table S6B. ADK and SnRK1 activities in ADK RNAi lines

	ADK activity			SnRK1 activity			t-test values	t-test values
	Mock	Dex	Fold change (Dex/Mock) Mean±SE	Mock	Dex	Fold change (Dex/Mock) Mean±SE	ADK activity	SnRK1 activity
dsADK-L4	469803	188962	0.400±0.009	52124	55138	1.045±0.056	4.19E-06	4.12E-01
dsADK-L6	732998	363729	0.496±0.006	70707	73624	1.034±0.042	2.29E-08	3.32E-01

Activities of ADK and SnRK1 in crude extracts from transgenic Arabidopsis lines dsADK-L4 and dsADK-L6 expressing ADK dsRNA from a dexamethasone (dex) inducible promoter are shown. Data were obtained from three independent experiments using pooled tissue from three plants, with two replicates each. Activity values (arbitrary units) were obtained by measuring signal intensity of ^{32}P labeled GST-SAMS (SnRK1 substrate) or 5'AMP (ADK product) by exposing PAGE gels or TLC plates, respectively, to a phosphorimager. Data are shown graphically in Figure 7B.

Table S6C. SnRK1 and ADK activities in SnRK1 over-expression lines

	SnRK1 activity	Fold change over WT (Mean±SE)	ADK activity	Fold change over WT (Mean±SE)	t test values SnRK1 activity	t-test values ADK activity
WT	11866	1±0.196	293369	1±0.083	-	-
S3	21560	1.817±0.233	349548	1.191±0.295	0.0301	0.3366
S5	31584	2.662±0.156	224633	0.766±0.107	0.0001	0.1009

SnRK1 and ADK activities in crude extracts from transgenic *N. benthamiana* lines S3 and S5 expressing SnRK1 from the constitutive 35S promoter are shown. Data were obtained from two independent experiments using two individual plants from each line and two replicate samples. Activity values (arbitrary units) were obtained by measuring signal intensity of ^{32}P labeled GST-SAMS (SnRK1 substrate) or 5'AMP (ADK product) by exposing PAGE gels or TLC plates, respectively, to a phosphorimager. Data are shown graphically in Figure 7C.

Table S6D. SnRK1 and ADK activities in SnRK1 antisense lines

	SnRK1 activity	Fold change over WT (Mean±SE)	ADK activity	Fold change over WT (Mean±SE)	t test values SnRK1 activity	t test values ADK activity
WT	36719	1±0.047	142632	1±0.060	-	-
AS-4	14784	0.402±0.090	1471023	9.417±2.052	0.0017	0.0119
AS-12	13056	0.355±0.067	1143831	7.441±0.409	0.0004	0.0002

SnRK1 and ADK activities in crude extracts from transgenic *N. benthamiana* lines AS-4 and AS-12 expressing antisense SnRK1 RNA from the constitutive 35S promoter are shown. Data were obtained from two independent experiments using two individual plants from each line and two replicate samples. Activity values (arbitrary units) were obtained by measuring signal intensity of ^{32}P labeled GST-SAMS (SnRK1 substrate) or 5'AMP (ADK product) by exposing PAGE gels or TLC plates, respectively, to a phosphorimager. Data are shown graphically in Figure 7D.
